# Cadherin Switch during EMT in Neural Crest Cells Leads to Contact Inhibition of Locomotion via Repolarization of Forces

**DOI:** 10.1016/j.devcel.2015.06.012

**Published:** 2015-08-24

**Authors:** Elena Scarpa, András Szabó, Anne Bibonne, Eric Theveneau, Maddy Parsons, Roberto Mayor

**Affiliations:** 1 Cell and Developmental Biology Department, University College London, Gower Street, London WC1E 6BT, UK; 2 Centre de Biologie du Développement–UMR5547, Centre National de la Recherche Scientifique and Université Paul Sabatier, Toulouse 31400, France; 3 Randall Division of Cell and Molecular Biophysics, Kings College London, London SE11UL, UK

## Abstract

Contact inhibition of locomotion (CIL) is the process through which cells move away from each other after cell-cell contact, and it contributes to malignant invasion and developmental migration. Various cell types exhibit CIL, whereas others remain in contact after collision and may form stable junctions. To investigate what determines this differential behavior, we study neural crest cells, a migratory stem cell population whose invasiveness has been likened to cancer metastasis. By comparing pre-migratory and migratory neural crest cells, we show that the switch from E- to N-cadherin during EMT is essential for acquisition of CIL behavior. Loss of E-cadherin leads to repolarization of protrusions, via p120 and Rac1, resulting in a redistribution of forces from intercellular tension to cell-matrix adhesions, which break down the cadherin junction. These data provide insight into the balance of physical forces that contributes to CIL in cells in vivo.

## Highlights

• Neural crest cells acquire contact inhibition of locomotion (CIL) during EMT
• An E- to N-cadherin switch controls CIL
• E-cadherin represses CIL by controlling Rac1-dependent protrusions via p120
• During CIL, forces are redistributed from intercellular junctions to cell matrix

## Graphical Abstract

**Figure:**
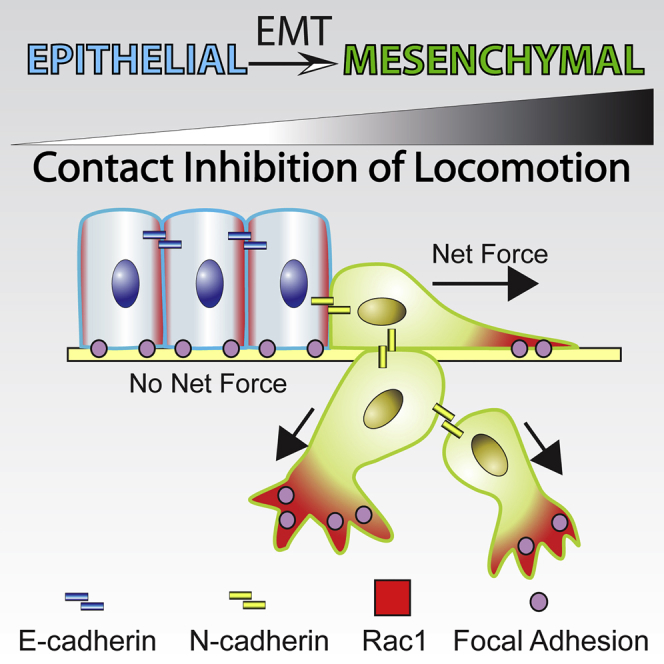

## Teaser

Cell-cell adhesions are transiently formed during contact inhibition of locomotion. Scarpa et al. show that an E- to N-cadherin switch controls CIL acquisition upon EMT. E-cadherin loss leads to repolarization of protrusions via p120 and Rac1, resulting in redistribution of forces from intercellular tension to cell-matrix adhesions, triggering junction disassembly.

## Introduction

More than 50 years ago, Abercrombie and Heaysman discovered that the direction of migration of chick heart fibroblasts cultured in vitro was modified by their interaction with other cells (Abercrombie and Heaysman, 1953). This process was defined as contact inhibition of locomotion (CIL). Its potential importance emerged as Abercrombie and colleagues showed that invasion of normal fibroblasts by malignant mesenchymal cells was linked to a modified CIL response, linking CIL to invasive metastasis (Abercrombie, 1979; Abercrombie and Ambrose, 1962; Abercrombie and Heaysman, 1954). More recently, CIL was shown to regulate the invasiveness of prostate malignant cells toward stromal fibroblast (Astin et al., 2010). Furthermore, the requirement of CIL in guiding complex migratory processes during embryonic development has been demonstrated in vivo for neural crest (NC) cells and macrophages (Carmona-Fontaine et al., 2008; Stramer et al., 2010). The molecular pathways underlying CIL remained poorly understood for decades. However, in both prostate cancer cells and *Xenopus* NC cells, the CIL response seems to rely on cell-cell contact-dependent signaling. In particular, Eph-Ephrin signaling has been found to be responsible for CIL in cancer cells (Astin et al., 2010), while in NC cells, activation of Wnt-PCP pathway leads to recruitment of Frizzled to the cell-cell contacts and activation of RhoA-ROCK, which is required for cell separation (Carmona-Fontaine et al., 2008). In addition, it has been suggested that cadherin-dependent cell-cell adhesion is required for CIL (Becker et al., 2013; Theveneau et al., 2010, 2013). During *Xenopus* neural crest-neural crest (NC-NC) and neural crest-placode (NC-PL) cell-cell interactions, N-cadherin is functionally required for CIL (Theveneau et al., 2010, 2013), and a classical cell adhesion complex formed by N-cadherin, p120, α-catenin, and β-catenin is transiently assembled upon these cell-cell interactions (Theveneau et al., 2010, 2013). However, both the NC-NC and the NC-PL junctions have a short half-life and eventually disassemble (Theveneau et al., 2013). Many pending questions remain. Why do certain cell types undergo CIL, whereas others cells do not? Why do some cell-cell interactions lead to the formation of a stable adherens junction while during CIL these junctions are transient?

Here we have used NC cells, a migratory embryonic stem cell population, to address these questions. We show that NC cells acquire CIL at the same time that they activate their epithelial-to-mesenchymal (EMT) program and start migrating. By comparing premigratory and migratory NC cells, we show that switching E- to N-cadherin during EMT is essential for CIL. We demonstrate that prior to EMT E-cadherin inhibits contact-dependent cell polarity via p120 and Rac1. Culturing NC on micropatterns, photoactivating different forms of Rac and measuring traction forces during CIL, we conclude that the cadherin switch leads to cell-cell junction breakdown by generating higher forces resulting from cell repolarization.

## Results

### CIL Is a Developmentally Regulated Property of NC Cells Acquired during EMT

*Xenopus* NC cells are an archetypical model for CIL, whose CIL response is well characterized, and it is essential for their directional migration in vivo and in vitro (Carmona-Fontaine et al., 2008; Moore et al., 2013; Theveneau et al., 2010).

**Figure 1:**
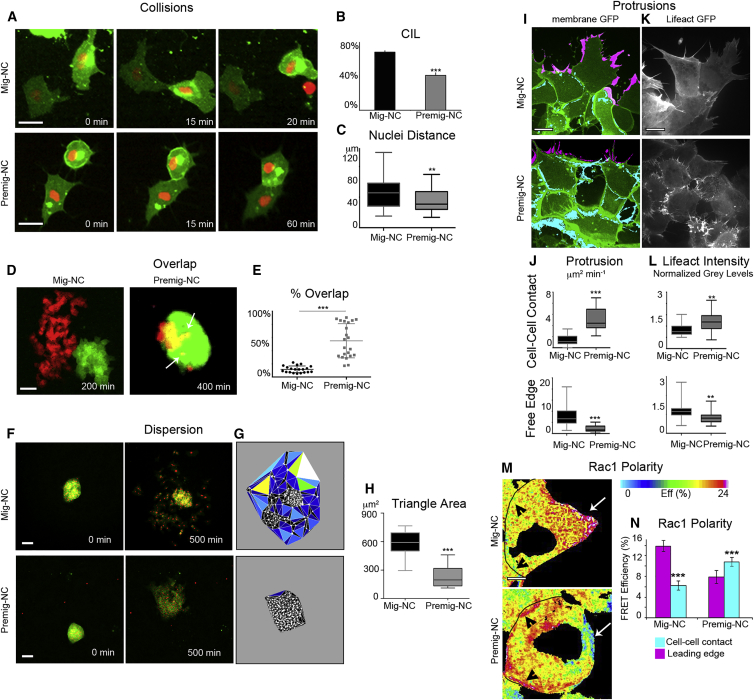
Migratory, but Not Premigratory, NC Exhibit CIL (A) Collisions of Mig-NC and Premig-NC. Scale bar is 20 μm. Time in minutes is indicated. Note that Mig-NC moves away from each other, while Premig-NC remains in contact. (B) Percentage of collisions displaying CIL (Mig-NC, n = 132, Premig-NC, n = 98), ^∗∗∗^α = 0.1%. (C) Distance between nuclei 30 min after collision (Mig-NC, n = 80, Premig-NC, n = 64), ^∗∗^p < 0.01. (D) CIL is analyzed by measuring the overlap between two NC explants, which is minimal for Mig-NC as they exhibit CIL. Scale bar represents 60 μm. (E) Percentage of overlap between explants (Mig-NC, n = 19, Premig-NC, n = 19), ^∗∗∗^p < 0.001. (F) Dispersion assay for Mig-NC and Premig-NC explants. Scale bar represents 50 μm. Note that Mig-NC disperses more efficiently than Premig-NC. (G) Cell dispersion was analyzed by measuring the area between neighbor cells (nuclei) at 500 min, color coded according to size of triangles. (H) Triangle area (Mig-NC, n = 10, Premig-NC, n = 23), ^∗∗∗^p < 0.001. (I) Protrusive activity of Mig-NC and Premig-NC. Maximal projection, free edge protrusions are labeled in magenta, and cell-cell contact protrusions are in cyan. Scale bar represents 10 μm. (J) Protrusion area per minute per cell obtained by subtraction between consecutive frames (Mig-NC, n = 45, Premig-NC, n = 80),^∗∗∗^p < 0.001. (K) Lifeact-GFP. Scale bar represents 10 μm. (L) Lifeact-GFP fluorescence intensity (Mig-NC, n = 12, Premig-NC, n = 15), ^∗∗∗^p < 0.001. (M) Spatial distribution of Rac1 FRET efficiency. Cell-cell junctions are outlined in black, with the free edge pointed to with an arrow; scale bar represents 5 μm. (N) Rac1 polarity (Mig-NC, n = 24, Premig-NC, n = 24), ^∗∗∗^p < 0.001. All box and whiskers charts are box/median ± 25^th^/75^th^ percentile. Whiskers are min/max value, and bar charts are mean ± SEM.

To investigate whether CIL is an intrinsic property of NC or whether it is acquired during NC development, we cultured *Xenopus laevis* premigratory NC (Premig-NC) before they undergo EMT and compared them with migratory NC (Mig-NC) after EMT has taken place. Nearly 80% of observed cell-cell collisions of Mig-NC showed typical CIL by forming a transient contact, stopping migration and moving away, while only 40% of Premig-NC collisions exhibited CIL (Figures 1A and 1B; Movie S1, collision assay) with most Premig-NC forming a stable contact and their nuclei remaining within a short cell-cell distance (Figure 1C). This differential behavior is not due to a difference in cell motility as the speed of migration is the same between Premig-NC and Mig-NC (Figures S1A and S1B). At the cell population level, CIL is known to prevent cell mixing, as has been shown in Mig-NC explants exhibiting CIL (Carmona-Fontaine et al., 2008). While our observations in Mig-NC explants confirm this result (Figures 1D and 1E), the Premig-NC intermingled readily indicating a lack of CIL (Figures 1D and 1E; Movie S1, overlap assay). At migratory stages, NC explants are known to undergo EMT in vitro (Kuriyama et al., 2014) and disperse due to CIL (Carmona-Fontaine et al., 2011; Woods et al., 2014). Such dispersion was observed in Mig-NC explants but not in Premig-NC (Figure 1F; Movie S1, dispersion assay), as shown by Delaunay triangulation analysis (Carmona-Fontaine et al., 2011) (Figures 1G and 1H). During CIL, cell protrusions are polarized via small GTPase activity leading to the formation of lamellipodia away from the cell contact in migrating NC clusters (Carmona-Fontaine et al., 2008; Theveneau et al., 2010). In Mig-NC explants, most protrusions pointed away from the cell contact and toward the free space, while in Premig-NC, most lamellipodia were beneath the cell-cell contacts, as shown by cell membrane (Figures 1I and 1J; Movie S1, protrusion analysis) and F-actin distribution (Figures 1K and 1L; Movie S1, LifeAct-GFP). Consistently, Rac1 activity in Mig-NC was high at the free edge (Figures 1M, top, white arrows, and 1N) and low at cell-cell contacts (Figures 1M, top, black arrowheads, and 1N), as detected by FRET (Itoh et al., 2002; Theveneau et al., 2010). Importantly, Rac1 activity in Premig-NC was reversed, being low at the free edge (Figures 1M, bottom, white arrows, and 1N) and high at cell-cell contacts (Figures 1M, bottom, black arrowheads, and 1N). Interestingly, the difference in protrusive activity between Premig-NC and Mig-NC, which is likely to be a consequence of the differential distribution of active Rac1, affected higher order features of CIL such as intermixing between cells (Figures S1C–S1F). High-resolution imaging of explant overlap assays shows that boundaries between differentially labeled Mig-NC clusters are significantly straighter (Monier et al., 2010) than in Premig-NC (Figures S1C and S1D). In addition, the duration of protrusions at the boundary (Figure S1E, arrowheads) was significantly higher in Premig-NC (Figures S1E and S1F), while Mig-NC tended to collapse protrusions upon contact (Figures S1E and S1F, arrows).In summary, these results show that NC cells acquire CIL at the time of their EMT.

### Analysis of Cell Junctions during CIL

**Figure 2:**
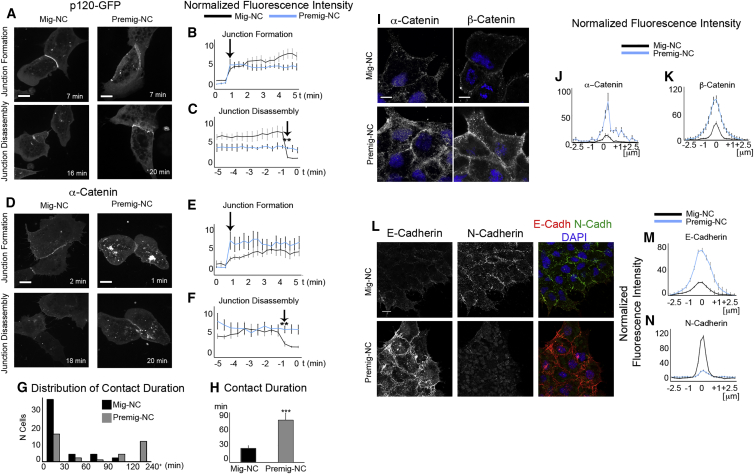
A Cadherin Switch Occurs during NC EMT (A–F) Assembly and disassembly of cell-cell junctions during collisions of Mig-NC or Premig-NC expressing p120-GFP (A) and α-catenin-GFP (D). Scale bars are 10 μm. Fluorescence intensity of p120-GFP (B) and α-catenin-GFP (E) at cell-cell contact are normalized to adjacent cytoplasm during first 5 min of collisions (p120-GFP: Mig-NC, n = 6, Premig-NC, n = 4; α-catenin-GFP: Mig-NC, n = 9, Premig-NC, n = 7). Normalized fluorescence intensity of p120-GFP (C) and α-catenin-GFP (F) at cell-cell contact during the last 5 min of cell-cell collisions. (G) Average contact duration for Mig-NC and Premig-NC (n = 43, Mig-NC, n = 30, Premig-NC), ^∗∗∗^p < 0.001. (H) Distribution of contact duration for Mig-NC and Premig-NC (n = 43, Mig-NC, n = 30, Premig-NC). (I) Immunostaining for α-catenin and β-catenin in Mig-NC and Premig-NC. Scale bars are 10 μm, and nuclear staining is DAPI. (J and K) Fluorescence intensity across cell-cell junctions normalized to fluorescence in adjacent cell cytoplasm (α-catenin: Mig-NC, n = 50; Premig-NC, n = 50) (β-catenin: Mig-NC, n = 50; Premig-NC, n = 50). (L) Double immunostaining for E- and N-cadherin in Mig-NC and Premig-NC. Scale bars represent 20 μm. (M and N) Normalized fluorescence intensity diagrams (Mig-NC, n = 74, Premig-NC, n = 74). All box and whiskers charts are box and median ± 25^th^/75^th^ percentile. Whiskers are min/max value, and bar charts are mean ± SEM.

The distinct behavior of Mig-NC and Premig-NC in response to cell-cell interactions might arise from differential dynamics of junction formation. To test this, we expressed p120-GFP or α-Catenin-GFP in Mig-NC or Premig-NC and imaged cell collisions with high time resolution. Expression of p120-GFP or α-Catenin-GFP did not per se affect the CIL response of Mig-NC (Figures S2A and S2B). Both Mig-NC and Premig-NC formed junctions containing p120 (Figures 2A–2C; Movie S2) and α-catenin (Figures 2D–2F; Movie S2) with similar dynamics. However, in Mig-NC, cell-cell contacts were rapidly disassembled while they persisted in Premig-NC (Figures 2C and 2F). Indeed, the duration of contact in Premig-NC was strongly increased when compared with Mig-NC (Figures 2G and 2H). Taken together, these findings suggest that Mig-NC is unable to stabilize their junctions. Based on this, we postulated that the composition of endogenous adherens junctions might be different in Mig-NC and Premig-NC. Indeed, α-catenin and β-catenin levels of immunostaining were higher in Premig-NC adhesions than in Mig-NC junctions (Figures 2I–2K). Second, we analyzed the expression of classical cadherins in Mig-NC and Premig-NC since cadherin switching has been observed during EMT in cancer cells and NC development in other organisms (Dady et al., 2012; Wheelock et al., 2008), and our data demonstrate that the acquisition of CIL correlates with EMT. We found that Mig-NC predominantly expressed N-cadherin, while Premig-NC expressed E-cadherin (Figures 2L–2N). The differential cadherin expression suggests that cadherin switching might be linked to the acquisition of CIL.

### E-Cadherin Suppresses CIL by Controlling Contact-Dependent Polarity of Rac1

**Figure 3:**
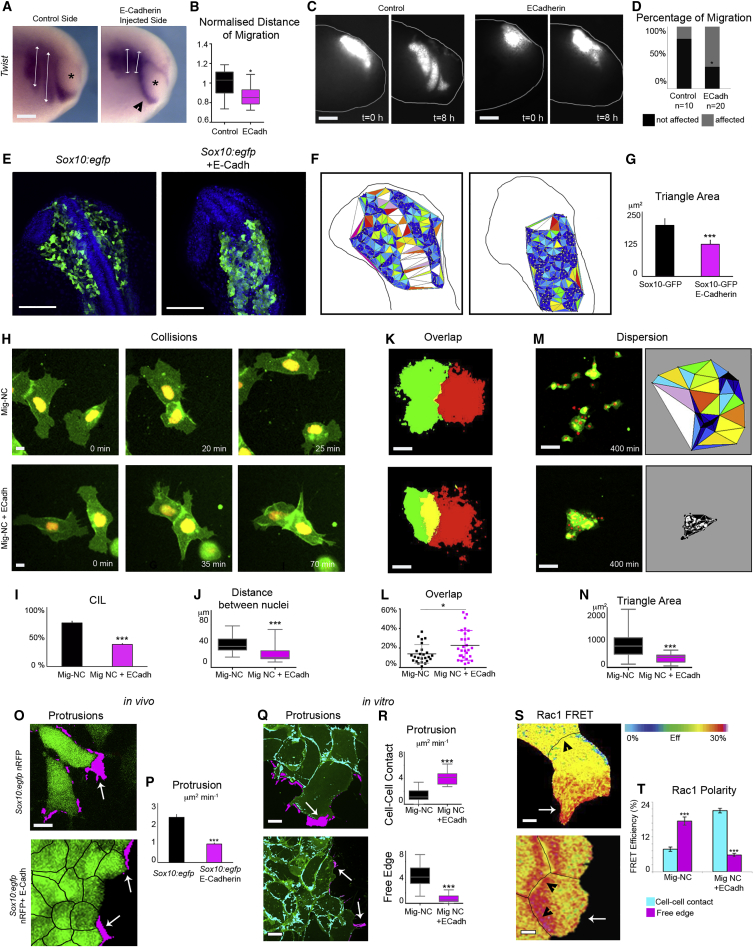
E-Cadherin Inhibits NC Migration In Vivo and CIL In Vitro (A) NC migration was analyzed in vivo by performing an in situ hybridization against the NC marker *Twist* of stage 25 *Xenopus laevis* embryo. Note the longer NC streams in control compared with E-cadherin expressing embryos. Asterisks are eye. Scale bar represents 200 μm. (B) Distance of migration for each stream. The injected side is normalized to the uninjected side (n = 19 embryos, ^∗^p < 0.05). (C) Fluorescently labeled WT or E-Cadh expressing NC grafted into WT embryos before (t = 0) and after (t = 8 hr) migration. Scale bars represent 250 μm. Note that inhibition of NC migration by E-cadherin is cell autonomous. (D) Percentage of migrating NC grafts (control, n = 10, E-Cadh, n = 20), ^∗^α = 5%. (E) Confocal projection of *sox10:egfp* zebrafish embryos injected with nuclear RFP (left) or nuclear RFP +E-Cadherin (right). Blue is DAPI. Green is GFP fluorescence. Note the dramatic inhibition in cell dispersion in E-cadherin-injected embryos. Scale bar represents 100 μm. (F) Cell dispersion was quantified by measuring the area of triangles formed by neighboring cells. Color-coded triangulation diagram for the images in (E). (G) Triangle area (*sox10:egfp*, n = 6, *sox10:egfp* +E-Cadh, n = 6), ^∗∗∗^p < 0.001. (H) Collisions of Mig-NC or Mig-NC+ECadh cells. Scale bar represents 10 μm. (I) Percentage of CIL (Mig-NC, n = 40, Mig-NC+E-Cadh, n = 29), ^∗∗∗^α = 0.1%. (J) Distance between nuclei, ^∗∗∗^p < 0.001. (K) Explant overlap assay, thresholded images. Scale bar represents 60 μm. (L) Percentage of overlap between explants (Mig-NC, n = 25, Mig-NC+E-Cadh, n = 28),^∗^p < 0.05. (M) Dispersion assay. Mig-NC and Mig-NC+ E-Cadh at 400 min (left) and color-coded triangulation diagram (right). Scale bar represents 50 μm. (N) Triangle area (Mig-NC, n = 28, Mig-NC+E-Cadh, n = 22). ^∗∗∗^p < 0.001. (O) Time lapse stills of living *sox10:egfp* NC cells in vivo. Free edge protrusions in magenta. Arrows represent protrusions. Scale bar represents 10 μm. (P) Quantitation of protrusion area per minute per cell in vivo (*sox10:egfp*, n = 72 cells, *sox10:egfp* +E-Cadh, n = 168 cells), ^∗∗∗^p < 0.001. (Q) Protrusive activity of Mig-NC and Mig-NC+E-Cadh. Maximum projection and free edge protrusions are in magenta. Cell-Cell contact protrusions are in cyan. Scale bar represents 10 μm. (R) Quantitation of Protrusion Area per minute per cell (Mig-NC n = 43, Mig-NC+E-Cadh n = 66), ^∗∗∗^p < 0.001. (S) Rac1 FRET efficiency. Cell-cell junctions are outlined in black. Scale bar represents 5 μm. (T) Rac1 FRET efficiency at cell-cell contact and at leading edge (Mig-NC, n = 24, Mig-NC+E-cadh, n = 24), ^∗∗∗^p < 0.001. All box and whiskers charts are as follows: box and median are ± 25^th^/75^th^ percentile. Whiskers are min/max value, and bar charts are mean ± SEM.

To explore whether the E- to N-cadherin switching is required for acquisition of CIL by migratory NC, we expressed E-cadherin ectopically in Mig-NC. As CIL is required for migration in vivo (Carmona-Fontaine et al., 2008), we analyzed the consequences of ectopic E-cadherin expression on NC migration. Overexpressing E-cadherin was sufficient to reduce the migration of NC cells in vivo in *Xenopus* embryos (Figures 3A and 3B). Furthermore, the effect of E-cadherin on NC migration is cell autonomous, as grafting E-cadherin expressing NC tissue in control embryos severely impaired migration compared to control grafts (Figures 3C and 3D). In line with our results in cultured *Xenopus* NC cells, in zebrafish, E-cadherin is expressed in premigratory NC, but not in migratory cells (Figure S3A). Importantly, ectopic expression of E-cadherin impaired NC migration and dispersion in vivo in zebrafish embryos (Figures 3E–3G). Observation of cell-cell collisions in vitro shows that E-cadherin expression reduces CIL compared with control Mig-NC (Figures 3H–3J; Movie S3, collision assay). Expression of E-cadherin does not affect the motility of single cells (Figure S3B), while it reduces the migration speed of cell doublets after collision (Figures S3C and S3D). The effect of E-cadherin on CIL was confirmed by overlap assays demonstrating that intermixing between Mig-NC cell clusters was increased by E-cadherin overexpression (Figures 3K and 3L). Accordingly, in line with in vivo observation in zebrafish embryos, ectopic E-cadherin strongly affected cell dispersion (Figures 3M and 3N; Movie S3, dispersion assay). Next we investigated the effect of E-cadherin expression on the polarity of cell protrusions. In vivo, migrating zebrafish NC cells were polarized and formed large protrusions at the free edge, whereas E-cadherin expressing cells formed little protrusions at the edge, remained rounded, and failed to delaminate (Figures 3O and 3P; Movie S3, Sox10:egfp). A similar situation was observed in vitro, as *Xenopus* Mig-NC produced Rac1-positive protrusions toward the free edge and few protrusions at cell-cell contacts (Figures 3Q–3T). This polarity was reversed in E-cadherin overexpressing Mig-NC (Figures 3Q–3T; Movie S3, protrusion analysis). To test whether these effects were specific to E-Cadherin or simply due to an overall increase of cell-cell adhesion strength, we overexpressed N-cadherin in Mig-NC and assessed the effect on cell collisions (Figure S3E), explant overlap assays (Figures S3F and S3G), and cell dispersion (Figures S3H and S3I). In contrast to the E-cadherin expression experiments, none of these assays were affected by N-cadherin overexpression, indicating an E-cadherin-specific effect on CIL and polarity. Because CIL has been reported to be dependent on N-cadherin (Theveneau et al., 2010, 2013) and ectopic expression of one cadherin may result in downregulation of another via competition for binding to p120 (Xiao et al., 2003), we tested whether expression of E-cadherin in Mig-NC might result in downregulation of N-cadherin levels. We found that E-cadherin did not decrease endogenous N-cadherin expression or other components of the cell adhesion complex, such as α- or β-catenin (Figures S4E–S4J). Taken together, these results strongly suggest that E-cadherin acts as a repressor of CIL in Mig-NC.

To further substantiate this, we performed E-cadherin loss-of-function experiments in Premig-NC, which do not exhibit CIL. To inhibit E-cadherin function, we used a morpholino oligonucleotide (MO) targeted against E-cadherin (Nandadasa et al., 2009) or an E-cadherin blocking antibody (5D3) (Theveneau et al., 2013). Explant overlap assays showed that intermixing between explants was reduced in Premig-NC following E-cadherin inhibition (Figures S3J and S3K). In addition, we assessed the dynamics of protrusions in Premig-NC injected with a control MO or E-cadherin MO. In control Premig-NC, protrusions were formed predominantly at cell-cell contacts and were small at free edges, while E-cadherin MO injected cells showed a reverted polarity (Figures S3L and S3M). Importantly, the effect of E-Cadherin knockdown was specific, as an epithelial-like polarity could be restored by expression of a morpholino-insensitive XE-cadherin mRNA (Figures S3L and S3M) (Nandadasa et al., 2009). To test whether the suppressive activity of E-cadherin on CIL was specific for NC cells, we analyzed another embryonic cell type, placodal cells (PLs), which are able to undergo heterotypic CIL when contacting Mig-NC (Theveneau et al., 2013). Epibrachial placodes express both E- and N-cadherin (Figure S3N) and do not display CIL when contacting one another (Figures S3O–S3Q). Importantly, E-cadherin knockdown significantly reduced intermixing between clusters of PL cells (Figures S3R and S3S) and induced an outward-directed protrusion polarity in PL cells explants (Figures S3T and S3U). These results suggest that ability of E-cadherin to inhibit CIL is not restricted to NC cells, but it is likely to be a more general phenomenon. Overall, our results indicate that E-cadherin acts as a repressor of CIL and its downregulation during EMT is a required step for acquisition of CIL in normal development.

To understand the mechanism through which E-cadherin inhibits CIL, we further analyzed whether E-cadherin levels affect the composition of Mig-NC cell-cell junctions. No qualitative difference was observed in the adhesion complex between Mig-NC and E-cadherin expressing Mig-NC, as both express α-catenin, β-catenin, and p120, although they were accumulated at higher levels in Mig-NC +E-cadherin explants (Figures S4A–S4D), thus suggesting a difference in biochemical interaction or in the dynamics of catenin complex recruitment between N- and E-cadherin. However, comparison of the N- and E-cadherin immunoprecipitations demonstrates that both cadherins interact with endogenous α- and β-catenins with comparable affinity (Figures S4K–S4N). These results suggest that E-cadherin does not affect CIL via the qualitative composition of the adhesion complex components, but may regulate CIL through an alternative mechanism. In Mig-NC, stability of endogenous N-cadherin-catenin complex is low due to endocytic recycling (Kuriyama et al., 2014). We assessed whether E-cadherin expression affected the mobility of the cadherin-catenin complex by performing FRAP for p120-GFP and α-catenin-GFP. The mobile fractions of p120-GFP and α-catenin-GFP decreased slightly but significantly upon E-Cadh expression (Figures S4O–S4Q) and the halftime of recovery increased significantly for both p120-GFP and α-catenin-GFP (Figure S4R). The effects observed in Mig-NC upon E-Cadh expression might be ascribed to a mild stabilization of the catenin complex protein dynamics.

### The Interaction between E-Cadherin and p120ctn Is Required to Suppress CIL

**Figure 4:**
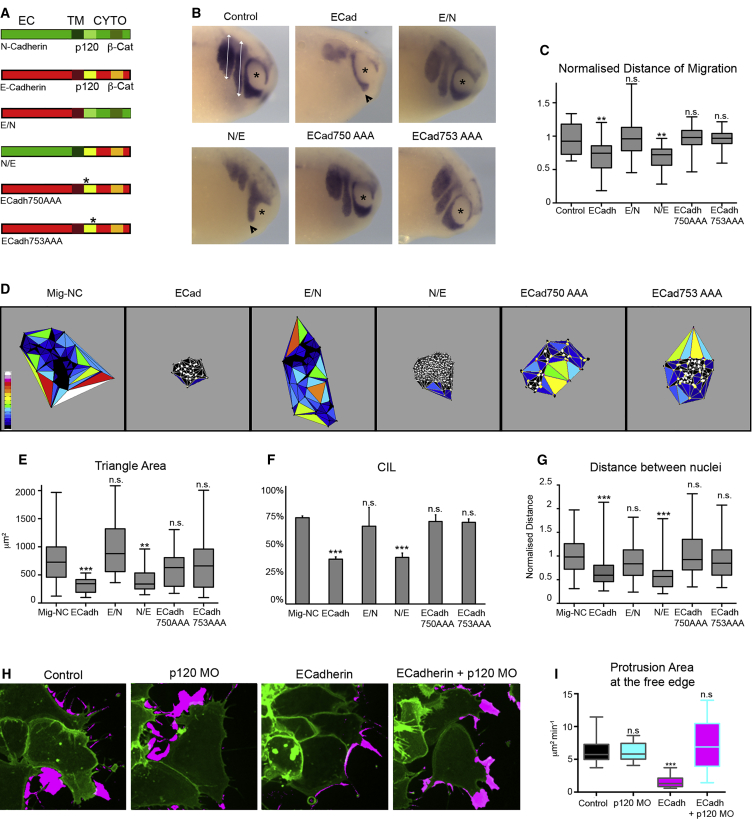
E-cadh-p120 Interaction Is Required to Promote Cell Repolarization (A) Diagram of N- and E-cadherin domain organization: EC domain, transmembrane domain (TM), and cytoplasmic domain (CYTO). E/N mutant, E-Cadh EC/N-Cadh CYTO. N/E mutant, N-Cadh EC/E-Cadh CYTO. Point mutations (750 GGG→AAA), (753 EED→AAA) in the juxtamembrane domain of E-Cadh are represented by the asterisk. (B) NC migration in vivo, in situ hybridization against the NC marker *Twist* at stage 25 X.L. embryos. Asterisks, eye; white lines, distance of NC migration. Scale bar represents 200 μm. (C) Distance of migration. Injected side normalized to uninjected side (control, n = 14, E-Cadh, n = 23, E/N, n = 20, N/E, n = 23, 750AAA, n = 10, 753AAA, n = 17), ^∗^p < 0.05, ^∗∗^p < 0.01. (D and E) Dispersion Assay triangulation diagrams (D) and triangle areas (E) at 400 min (Mig-NC, n = 28, E-Cadh, n = 22, E/N, n = 24, N/E, n = 19, 750AAA, n = 31, 753AAA, n = 27), ^∗^p < 0.05, ^∗∗∗^p < 0.01, ^∗∗∗^p < 0.001. (F) Percentage of CIL (Mig-NC, n = 105, E-Cadh, n = 71, E/N, n = 80, N/E, n = 55, 750AAA, n = 50, 753AAA, n = 60), ^∗∗∗^α = 0.1%. (G) Distance between nuclei 30 min after collision, ^∗∗∗^p < 0.001. (H) Protrusive activity of Mig-NC and Mig-NC+ECadh upon p120 knockdown. Time-lapse stills of a maximal projection are shown, and free edge protrusions are labeled in magenta. Scale bar represents 10 μm. (I) Quantitation of protrusion area per minute at cluster free edge per cell by subtraction analysis (control, n = 43, p120-MO, n = 50, E-Cadh, n = 58, E-cadh+p120 MO, n = 69), ^∗∗∗^p < 0.001. All box and whiskers charts are as follows: box and median are ± 25^th^/75^th^ percentile. Whiskers are min/max value, and bar charts are mean ± SEM.

We then sought to identify which functional domain of E-cadherin inhibits CIL. N- and E-cadherin are single pass transmembrane proteins, with an extracellular (EC) domain mediating *cis*- and *trans*-homophilic interactions, a transmembrane domain (TM), and a cytoplasmic domain with a direct binding site for p120 and β-catenin at the juxtamembrane and C-term regions respectively (Figure 4A). We generated two chimeric mutants by exchanging the EC domains of E- and N-cadherin (Figure 4A). In addition, since we observed a change in Rac1 activity upon E-cadherin expression and because p120 is involved in activating Rac1(Goodwin et al., 2003; Wildenberg et al., 2006), we abolished E-cadherin-p120 interaction by using two p120 uncoupled E-cadherin mutants (Ciesiolka et al., 2004): E-cadherin750AAA and E-cadherin753AAA (Figure 4A). Double immunostaining with N-cadherin and E-cadherin antibodies reactive against their respective EC domain confirmed that mutants were expressed and correctly localized at cell-cell junctions (Figure S5A). First, we expressed the WT, chimeric, or point mutant E-cadherin in embryos and compared their effects on NC migration in vivo (Figures 4B and 4C). Our data revealed that the N/E chimeric mutant was the only mutant that mimicked E-cadherin overexpression by reducing NC migration in vivo, indicating that the effect of E-cadherin requires the cytoplasmic domain and that this domain needs to interact with p120. Furthermore, dispersion (Figures 4D and 4E) and collision assays (Figures 4F and 4G) showed that the E-cadherin cytoplasmic domain and its interaction with p120 are also required to inhibit cell dispersion and CIL.

How does the E-cadherin-p120 signaling impact on polarity and CIL? FRET analysis of total Rac1 activity shows that Mig-NC+E cadherin exhibit higher Rac1 activity than control cells or cells expressing the p120 uncoupled E-cadherin mutants (Figure S5B), indicating that E-cadherin promotes Rac1 activity in NC cells via p120. We confirmed the importance of E-cadherin-p120 interaction by knocking down endogenous p120 in Mig-NC or Mig-NC + E-cadherin using a p120 MO (Ciesiolka et al., 2004) and by assessing the polarity and dynamics of protrusion formation (Movie S4). Importantly, p120 knockdown did not per se affect the polarity and size of protrusions (Figures 4H and 4I) but was sufficient to rescue the reduction in protrusive activity due to E-cadherin ectopic expression (Figures 4H and 4I). In addition, knockdown of p120 in premigratory NC cells, which express endogenous E-cadherin, induced a mesenchymal-like protrusion polarity with large protrusions at the cells’ free edge and little protrusive activity at the cell-cell contact (Figures S5C and S5D). Importantly, the effect of p120 knockdown was specific, as an epithelial-like polarity could be restored by expression of a morpholino-insensitive Xp120 mRNA (Figures S5C and S5D) (Ciesiolka et al., 2004). In conclusion, these results show that E-cadherin cytoplasmic domain signals via p120 to activate Rac1 at NC cell-cell junctions and leads to suppression of CIL and altered NC migration behavior in vivo.

### Repolarization of Protrusions Triggers Cell-Cell Junction Breakdown during CIL

**Figure 5:**
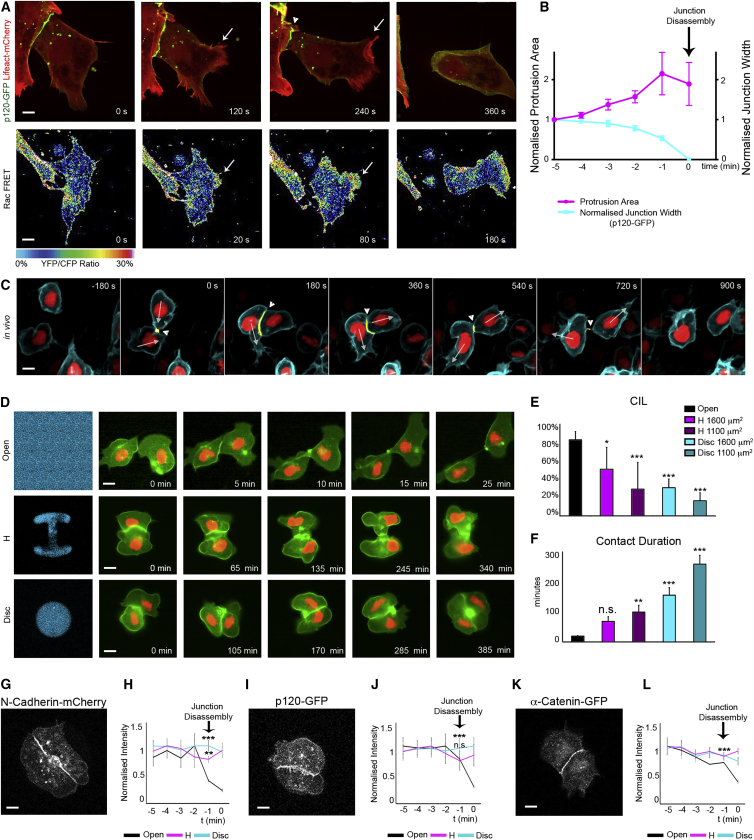
Repolarization Is Required to Promote Junction Disassembly (A) Time lapse stills of junction disassembly in a Mig-NC cell-cell collision. (Top) cells expressing p120-GFP and Lifeact-Cherry. Scale bar represents 5 μm. (Bottom) heatmap stills of Raichu-Rac1 FRET. Scale bar represents 7.5 μm. (B) Protrusion area and junction width over time. Junction disassembly occurs at t = 0. Cell-cell junctions were identified by p120-GFP (n = 11 cell-cell collisions), Spearman correlation coefficient r = −0.943, ^∗^p = 0.017. (C) Time lapse stills of cell-cell contact disassembly in colliding NC cells in vivo in *Sox10:H2BmCherry/GFP-GPI* zebrafish embryos. Arrows, direction of movement; arrowheads, cell-cell contact. Scale bar represents 10 μm. (D) Time lapse photographs of confined cells. Mig-NC labeled with membrane GFP and nuclearRFP (nRFP) cultured on uniform or H-shaped or disc-shaped micropatterns of fibronectin (Fn-650). Scale bar represents 10 μm. (E) Percentage of CIL (freely migrating [FM], n = 139, H, 1,600 μm^2^, n = 61, H, 1,100 μm^2^, n = 35, disc 1,600 μm^2^, n = 34, disc 1,100 μm^2^, n = 22), ^∗^α = 5%, ^∗∗∗^α = 0.1%. (F) Duration of cell-cell contact, ^∗∗^p < 0.01, ^∗∗∗^p < 0.001. (G, I, and K) Time-lapse stills of Mig-NC confined on a disc micropattern expressing N-cadherin-cherry (G), p120-GFP (I), α-catenin-GFP (K). Scale bar represents 5 μm. (H, J, and L) Fluorescence intensity over time for N-Cadherin-Cherry (H), p120-GFP (J), α-catenin-GFP (L). N-cadherin-cherry FM, n = 7, H, n = 4, disc, n = 6; p120-GFP FM, n = 7, H, n = 4, disc, n = 8; α-catenin-GFP FM, n = 9, H, n = 4, disc, n = 4, ^∗∗^p < 0.01. All box and whiskers charts are as follows: box and median are ± 25^th^/75^th^ percentile. Whiskers are min/max value, and bar charts are mean ± SEM.

Based on these data, we reason that the repolarization of protrusions away from the cell contact might be a causal factor in promoting the disassembly of the cell-cell junction that occurs during CIL. To support causality, we analyzed the temporal sequence of cell-cell junction disassembly and lamellipodial protrusion formation in collisions of Mig-NC, using p120-GFP and lifeact-Cherry to identify cell-cell junctions and lamellipodial protrusions respectively (Figure 5A, top; Movie S5). We found that new protrusions formed away from the cell contact while cell-cell adhesion complexes were still present (Figures 5A, arrows, and 5B). Moreover, protrusion area and junction width inversely correlated during CIL of Mig-NC (Spearman r = −0.9426, ^∗^p = 0.017). In addition, ratiometric Rac1 FRET imaging of live Mig-NC collisions (Figure 5A, bottom; Movie S5) demonstrates that active Rac1 increases opposite to the cell contact upon collision when the cells are still in contact. Consistent with our observations in cultured NC cells, imaging of cell-cell collisions occurring in vivo between zebrafish cranial NC cells shows that repolarization of protrusions occurs before the breakdown of the cell-cell contact (Figure 5C). These observations show that repolarization of protrusions opposite of the cell-cell contact site precede and therefore could promote junctional disassembly during CIL.

To address this hypothesis, we inhibited the formation of new protrusions in Mig-NC by restricting them on H-shaped or circular-shaped micropatterns of two different sizes (Tseng et al., 2012) and compared their ability to separate and undergo CIL with the same ability of cells without confinement. CIL, apparent in freely migrating Mig-NC (Figure 5D, top), was significantly decreased in cells plated on micropatterns where cells maintained cell-cell contacts (Figures 5D, middle and bottom, 5E, and 5F). These effects were even more evident on smaller micropatterns (Figures 5E and 5F; Movie S6, left column). Cell-cell junctions in cells under confinement were maintained throughout, as evidenced by the continued presence of junctional markers N-cadherin-Cherry, p120-GFP, and α-catenin-GFP, while junctions were disassembled between unconstrained cells (Figures 5G–5L; Movie S6, center and right columns). Therefore, we conclude that repolarization of protrusions is required for junction disassembly during CIL.

### Protrusion Repolarization via Rac1 Is Sufficient to Trigger Cell Separation during CIL

**Figure 6:**
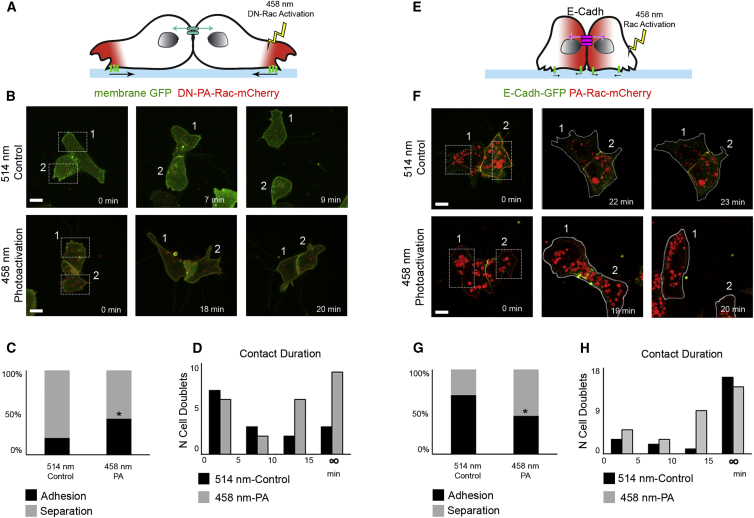
Inducing Protrusion Repolarization by Rac1 Photoactivation Is Sufficient to Trigger CIL and Junction Disassembly (A) DN-PA-Rac1 was photoactivated at the protrusions of Mig-NC cells. (B) Stills of Mig-NC doublets expressing DN-PA-Rac-Cherry. Illumination is shown in boxed areas with 514 nm control wavelength (top) or with 458 nm wavelength (bottom). Scale bar represents 10 μm. (C) Percentages of adhesion and separation upon Photoactivation in DN-PA-Rac-Cherry Mig-NC (514, n = 15, 458, n = 21). (D) Histogram of contact duration upon photoactivation in DN-PA-Rac-Cherry expressing Mig-NC. (E) PA-Rac1 was photoactivated at the edge of Premig-NC cells. (F) Stills of E-Cadh-GFP/PA-Rac-Cherry expressing Mig-NC doublets. Illumination is shown of boxed areas with 514 nm control wavelength (top) or with 458 nm wavelength (bottom). Scale bar represents 10 μm. (G) Percentages of adhesion and separation upon Photoactivation in E-Cadh-GFP/PA-Rac-Cherry Mig-NC (514 nm, n = 24, 458 nm, n = 31 cells), ^∗^α = 5%. (H) Histogram of contact duration in E-Cadh-GFP/PA-Rac-Cherry Mig-NC upon photoactivation. All box and whiskers charts are as follows: box and median are ± 25^th^/75^th^ percentile. Whiskers are min/max value, and bar charts are mean ± SEM.

Our previous experiments indicate that polarized protrusions are necessary for CIL. To confirm this conclusion and further test whether repolarization of protrusion upon collision is sufficient to drive CIL, we proceed to locally inhibit or activate Rac1 using different photoactivatable forms of Rac1 (Wu et al., 2009) (Figures 6A and 6B). We first verified the efficiency of Rac1 photoactivation in NC by illuminating single cells with control (control-514nm) or photoactivating wavelengths (PA-458nm) and measuring protrusion area in the illuminated box over time (Figure S6A; Movie S7). Only the PA-458nm was able to induce protrusions (Figure S6B) in PA-Rac expressing cells or to trigger protrusion collapse in DN-PA Rac expressing cells (Figures S6E and S6F; Movie S7). Consistently with what we observed in cell confinement experiments (Figures 5D–5K), blocking protrusion formation in Mig-NC doublets by using a dominant-negative-PA-Rac1(Wu et al., 2009) (DN-PA-Rac; Movie S7) prevented the separation of cells (Figures 6A–6D; Movie S7).

We then employed the PA-Rac1 to induce protrusion repolarization in E-cadherin expressing NC cell doublets (Figure 6E). Illumination of the free edges of Mig-NC+ECadh-GFP doublets with control-514nm (Figure 6F, top) did not result in new protrusions, and cells maintained their cell-cell junction. Illumination with PA-458nm, however, resulted in cell repolarization (Figure 6F, bottom) and an increased rate of cell separation (Figures 6G and 6H; Movie S7); this separation was not due to non-specific downregulation of junctional E-cadherin caused by laser illumination (Figures S6C and S6D; Movie S7). Taken together, these results strongly suggest that formation of new protrusions opposite to the cell contact is necessary and sufficient to promote junction disassembly.

### E-Cadherin Impairs CIL by Perturbing the Distribution of Forces in Mig-NC

**Figure 7:**
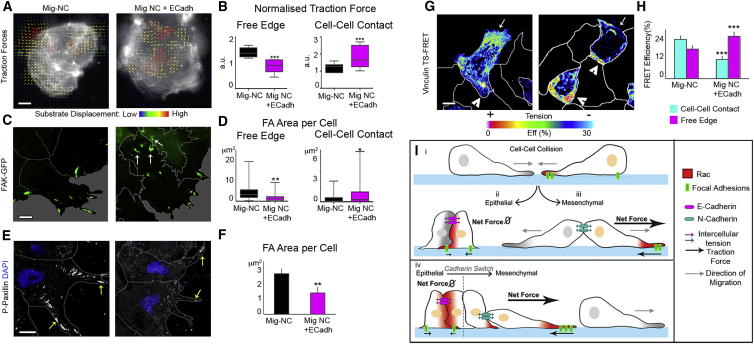
E-Cadherin Impairs CIL by Perturbing the Distribution of Forces in Mig-NC (A) Traction force microscopy superimposed to membrane RFP. Arrows show the magnitude and direction of bead displacement. Scale bar represents 20 μm. (B) Normalized TF at free edge (left) and cell-contacts (right) (Mig-NC, n = 9, Mig-NC+E-Cadh, n = 7), ^∗∗∗^p < 0.001. (C) Stills of Mig-NC and Mig-NC+E-Cadh explants expressing FAK-GFP. Cell borders are outlined, and scale bar represents 5 μm. Note that E-cadherin leads to the formation of FA at the cell-cell contact (arrows). (D) FA area per cell at free edge (left) and cell-contacts (right) (Mig-NC, n = 32, Mig-NC+E-Cadh, n = 56 cells), ^∗^p < 0.05, ^∗∗^p < 0.01. (E) P-paxillin immunostaining in Mig-NC and Mig-NC+ECadh. Arrows show FA at the free edge. Cell borders are outlined, and scale bar represents 10 μm. Nuclear staining is DAPI. (F) FA area per cell (Mig-NC, n = 10, Mig-NC+E-Cadh, n = 10 explants), ^∗∗^p < 0.01. (G) Spatial distribution of tension in Mig-NC and Mig-NC+E-Cadh clusters measured by Vinculin-TS FRET. In Mig-NC, tension is high at leading edge (arrow) and inhibited at cell-cell contact (arrowheads); in Mig-NC+E-Cadh, tension distribution is opposite. Cell borders are outlined, and scale bar represents 5 μm. (H) Vinculin-TS FRET efficiency at cell-cell contact and leading edge (n = 24 cells, Mig-NC, n = 24 cells, Mig-NC+E-Cadh), ^∗∗∗^p < 0.001. All box and whiskers charts show the following: box and median are ± 25^th^/75^th^ percentile. Whiskers are min/max value, and bar charts are mean ± SEM. (I) Model of CIL. (i) Cell collision is shown. Two possible outcomes exist, depending if cells are epithelial (ii) or mesenchymal (iii). (ii) Epithelial cells stabilize their junctions after collision. At the cell contact, Rac is activated, FAs are formed, and traction forces are generated. As there is no polarity on traction forces, the net forces is zero. (iii) Mesenchymal cells dissemble their junctions during CIL. At the cell contact, Rac is inhibited, FAs are disassembled, and traction forces are polarized at the free edge. (iv) E- to N-cadherin switch during EMT leads to CIL response.

Based on this evidence, we postulate that cells move away from each other during CIL because forces generated by the polarized protrusions give rise to stress sufficient to overcome the tensile strength of cell-cell adhesion sites, and this subsequently acts to pull the cells apart. Traction force microscopy applied to Mig-NC explants exhibiting polarized protrusions revealed that major forces are localized to the cluster’s edge and are oriented inward (Figure 7A). In contrast, Mig-NC+E-cadherin explants that do not display polarized protrusions exhibit randomly oriented traction forces in the middle of the clusters and significantly lower traction at the free edges compared with Mig-NC explants (Figures 7A and 7B). Since the size of focal adhesions (FAs) reportedly correlates with the traction force generated (Trichet et al., 2012), we analyzed the distribution, size, and dynamics of FAs by expressing FA kinase (FAK)-GFP (Figures 7C and 7D) or by immunostaining against Phospho-Paxillin (Figures 7E and 7F). Importantly, expression of FAK –GFP mRNA did not affect FA size or distribution when compared with endogenous Phospho-Paxillin (Figures S7A–S7C). Mig-NC explants show large and dynamic FAs distributed in a highly polarized fashion toward the free protruding edge (Figure 7C, left). By overexpressing E-cadherin, however, the number, size, and dynamics of FAs were reduced (Figures 7C–7F and S7D–S7F). Importantly, FA numbers were also reduced at the free edge in cells overexpressing E-cadherin (Figures 7E, arrows, and 7F). To confirm our observations on traction forces, we measured tension using a Vinculin tension sensor (Vinculin-TS) FRET probe (Grashoff et al., 2010; Kuriyama et al., 2014). In Mig-NC, tension across vinculin appeared to be high at free cell edges, where most FAs are present, whereas Mig-NC+E-cadherin cells showed a strong reduction in tension at the free edge (Figures 7G and 7H). Taken together, these data indicate that during EMT there is a dramatic repolarization of forces consistent with Mig-NC cells undergoing CIL and breaking down the junction, as the traction forces pull them apart.

## Discussion

These results support a model for CIL in which a transient adhesion complex is disassembled by polarized forces that break the cell junction. In cells without CIL, like epithelial Premig-NC or in Mig-NC overexpressing E-cadherin, Rac1 activity and FAs are polarized toward the cell-cell junction, and protrusions away from the contact are small, leading to smaller traction forces at the free edge counterbalanced by E-cadherin cell-cell junctions (Figure 7Iii). On the other hand, in cells that undergo CIL, such as mesenchymal Mig-NC that lacks E-cadherin, this polarity is reversed with highly polarized Rac1 activity, protrusions, and FAs formed away from the cell contact and high traction forces over the substrate, which are not counterbalanced by the N-cadherin intercellular junctions that eventually disassemble (Figure 7Iiii). We show that E-cadherin works as suppressor of CIL, whereas N-cadherin promotes it. Importantly, the E- to N-cadherin switch is a normal step of EMT. The loss of E-cadherin observed during EMT leads to cell polarization as described above, breaking the cell junction and contribution to cell dissemination of NC during normal development, and eventually of cancer cells during metastasis (Figure 7Iiv). We propose that CIL should be considered an additional characteristic of EMT.

We observed that E-cadherin knockdown in Premig-NC cells display a mesenchymal-like polarity even if they do not express N-cadherin at this developmental stage. However, other molecules involved in CIL such as Cadherin-11 and Frizzled-7 are expressed in NC cells at early stages (Becker et al., 2013; De Calisto et al., 2005; Kashef et al., 2009) and might be involved in promoting Premig-NC protrusive activity.

To understand whether the inhibition of cell separation driven by E-cadherin might be imputable to stronger cell-cell adhesion, we characterized the composition of E- and N-cadherin junctions in NC cells. Our results show that both E- and N-cadherin are able to organize an adhesion containing the junction components p120, β-catenin, and α-catenin, although in both premigratory and E-cadherin overexpressing NC cells the recruitment of catenins at the cell-cell junction is increased. However, analysis of the ability of E- or N-cadherin to biochemically interacts with β-catenin and α-catenin shows no significant difference in affinity of the two cadherins to the complex. FRAP analysis of p120 and β-catenin suggests that E-cadherin exerts a mild but significant effect on junctional stability in NC, which could explain the greater accumulation of adhesion proteins in the E-cadherin junction observed here. How this greater stability of the junctional complex is translated into absence of cell polarization remains unknown. Furthermore, it is currently unclear whether the strength of E- and N-cadherin-based junctions is different. Indeed, in vitro studies of analytical ultracentrifugation show that the homophilic binding affinity of N-cadherin EC domain is approximately 4-fold higher than for E-cadherin (Katsamba et al., 2009), while dual pipette separation studies performed in cells in suspension suggest the E-cadherin junction to be stronger than the N-cadherin one (Chu et al., 2004). Our findings suggest that the intracellular domain of E-cadherin rather than the EC adhesive domain is responsible for the suppression of CIL we observe. Whether the transmembrane domain of E-cadherin carries additional functions, as recently reported for VE-cadherin (Coon et al., 2015), still remains to be addressed. In contacting cells without CIL behavior, E-cadherin inhibits the formation of outward protrusions by controlling the distribution of active Rac1 in a p120-dependent manner. Whether p120 regulates Rac1 activity directly via a Rac GEF (Noren et al., 2000; Valls et al., 2012) or whether it acts on Rac1 indirectly by controlling integrin activation in the vicinity of the cell-cell contact (Ouyang et al., 2013) remains to be investigated. We have shown that, in the absence of E-cadherin mediated inhibition of protrusion repolarization, cell separation during CIL is driven by such protrusions. Whether the forces generated by the newly formed protrusions are transmitted directly to the cell-cell junction or whether these forces are necessary to generate a “trailing back” environment at the junction (Houk et al., 2012; Martin et al., 2014) remains unknown. Taken together, our findings suggest that the disassembly of cell-cell junctions taking place during CIL relies on a disproportion between intracellular tensions and traction forces exerted on the ECM by the repolarizing cells rather than on a weakening of cell-cell adhesion upon cadherin switching. Interestingly, it has been reported that EMT-inducing growth factors such as hepatocyte growth factor (HGF) do not alter the strength of E-cadherin cell-cell adhesions in epithelial cells (de Rooij et al., 2005; Hoj et al., 2014), but induce cell scattering by promoting formation of FAs on ECM; alteration of the ability of cells to exert traction forces on the ECM by using compliant substrates impairs scattering (Hoj et al., 2014), while stiffer substrates promote EMT (de Rooij et al., 2005). In addition, direct measurement of traction forces during Snail-induced EMT in epithelial cells shows that mesenchymal cells exert higher traction forces on the ECM (McGrail et al., 2015).

Importantly, live imaging of chick NC EMT shows that the adherens-junction containing apical tail is ruptured during delamination from the neural tube, thus suggesting that cell-cell junctions might be broken down as a consequence of tractional forces exerted by the delaminating NC cells (Ahlstrom and Erickson, 2009). Recent observations of CIL of hemocytes in *Drosophila* embryos shows an increase in tension across the cell contact (Davis et al., 2015), which is consistent with our observations; however, no analysis of how this tension is generated or the eventual contribution of cell polarization to disassemble of the adhesion complex was performed in that work (Davis et al., 2015; Roycroft and Mayor, 2015).

In conclusion, our study suggests a molecular mechanism linking two processes, EMT and CIL, leading to cell dissociation and cell dispersion. The generality of these processes raises the possibility that a wider range of cell types (i.e., metastatic cancer cells, other embryonic cells) undergoing similar qualitative changes of their cadherin repertoire might acquire CIL as part of their progression through EMT, contributing to disease progression or developmental morphogenesis.

## Experimental Procedures

### Microinjection and Embryology

*Xenopus laevis* embryos were microinjected as previously described (Carmona-Fontaine et al., 2008). For in vitro experiments, explants were dissected either at stage 15 or at stage 19 (Nieuwkoop and Faber) and plated on a fibronectin-coated dish as described in (Theveneau et al., 2010) or on a fibronectin micropatterned coverslip (CYTOO). For in vivo experiments, embryos were fixed at stage 25 to perform Twist in situ hybridization. Grafts of NC cells were performed at stage 16. Transgenic *sox10:egfp* (Carney et al., 2006) was maintained according to standard procedures. *sox10:egfp* was used to analyze NC migration in vivo (Carney et al., 2006). Embryos were processed as previously described (Matthews et al., 2008). Animal licenses were approved by the Home Office and University College London.

### Collision Analysis and Invasion Assays

For single-cell collision assays, NC cells were briefly dissociated in Ca^2+^/Mg^2+^-free Danilchick medium (Theveneau et al., 2010). CIL was assessed by counting cell separation events and distance between nuclei 30 min after contact initiation (Scarpa et al., 2013; Theveneau et al., 2013). Invasion assays were performed as previously described (Carmona-Fontaine et al., 2008; Theveneau et al., 2010).

### Cell Dispersion

NC cells from embryos injected with H2B-mCherry were imaged for 10 hr. To analyze dispersion, the Delaunay triangulation algorithm was deployed (Carmona Fontaine et al., 2011). This algorithm connects every cell to its closest neighbor, building a network of triangles and retrieving the area of each triangle. Delaunay triangulation is publicly available as an ImageJ plugin.

### FRET Imaging

For Ratiometric FRET, confocal imaging was carried out with a Nikon A1R laser scanning microscope. CFP and YFP were excited with 440 diode and 514 nm Argon ion laser lines, respectively, and detected through 470–500 nm bandpass and 530 nm longpass filters. FRET was detected by excitation of CFP and collection of emission with 530 nm longpass filters. Movies were corrected for bleedthrough between channels prior to background subtraction. Data were analyzed using the ImageJ RiFRET plugin (Roszik et al., 2009). For Acceptor Photobleaching, imaging was performed as previously described (Matthews et al., 2008).

### Immunostaining and Antibodies, Antisense MOs

Immunostaining of *Xenopus* NC was performed as previously described (Moore et al., 2013). See Supplemental Information for antibodies used. E-cadherin MO and p120 MO were purchased from Gene Tools and were used as previously described (Ciesiolka et al., 2004; Nandadasa et al., 2009).

### Preparation of Embryo Lysates and Immunoprecipitation

Embryos were lysed in lysis buffer (100 mM NaCl, 50 mM Tris-HCl, 1% Triton X-100) supplemented with the antipain, leupeptin, pepstatin, and phenylmethylsolfonyl fluoride (PMSF) (Sigma) at 10 μg/ml each. Immunoprecipitation was performed as described in (Gai et al., 2011). Samples were analyzed by SDS-PAGE.

### Traction Forces

The preparation of the polyacrylamide hydrogels containing fluorescent beads was adapted from previously published protocols (Dembo and Wang, 1999; Wang et al., 2002) and performed as previously described (Theveneau et al., 2013). Traction force measurements were performed as previously described (Lin et al., 2010).

### Photoactivation

Photoactivation of PA-Rac and DN-PA-Rac was performed as previously described (Wang et al., 2010; Wu et al., 2009).

### Statistical Analysis

Comparison of percentages was performed using contingency tables as described previously (Carmona-Fontaine et al., 2008). Normality of data sets was tested using Kolmogorov-Smirnov’s test, d’Agostino, and Pearson’s test using Prism6 (GraphPad). Data sets following a normal distribution were compared with Student’s t test (two-tailed, unequal variances) in Excel or a one-way ANOVA with a Dunnett’s multiple comparisons post-test in Prism6 (GraphPad). Data sets that did not follow a normal distribution were compared using Mann-Whitney’s test or a non-parametric ANOVA (Kruskal-Wallis with Dunn’s multiple comparisons post-test) using Prism6 (GraphPad). Cross-comparisons were performed only if the overall p value of the ANOVA was < 0.05.

## Supplemental Information

Document S1. Supplemental Experimental Procedures and Figures S1–S7

Movie S1. CIL Is a Developmentally Regulated Property of NC Cells, Related to Figure 1 (Collision assay) This is an example of Mig-NC collisions in which cells undergo CIL (left) and of Premig-NC cells forming a stable contact (right). Magenta is nuclear-mCherry. Green is membrane GFP. Frame delay is 5 minutes, and magnification is ×20. (Overlap assay) This is an example of Mig-NC explant overlap assay in which explants undergo CIL and do not overlap (left) and of Premig-NC, in which explants overlap and cells intermingle (right). Magenta is Rhodamine Dextran. Green is fluorescein-dextran. Frame delay is 5 minutes. Magnification is ×10. (Dispersion assay) Mig-NC explants undergo EMT and disperse (left), while Premig-NC does not disperse (right). Magenta is nuclear-mCherry. Green is membrane GFP. Frame delay is 5 minutes. Magnification is ×10. (Protrusion analysis) Mig-NC are strongly polarized and produce protrusions directed outward (arrow, left), while Premig-NC protrusions are not polarized (arrow, right) and produce protrusions at cell-cell contact sites (arrowhead). Magenta is nuclear-mCherry. Green is membrane GFP. Frame delay is 10 seconds. Magnification is ×60. (Lifeact-GFP) Mig-NC are strongly polarized and produce actin-rich protrusions directed outward (arrow, left), while Premig-NC protrusions are not polarized (arrow, right) and produce actin-rich protrusions at cell-cell contacts (arrowhead). Frame delay is 10 seconds, and magnification is ×60.

Movie S2. Different Dynamics of Junction Disassembly in Migratory and Premigratory NC Cells, Related to Figure 2 An example of Mig-NC collisions in which cells undergo CIL (left) and of Premig-NC, cells forming a stable junction (right). (Top) p120-GFP (green). (Bottom) α-catenin-GFP (green). Magenta is nuclear-mCherry. Frame delay is 20 seconds. Magnification is ×60.

Movie S3. E-Cadherin Suppresses CIL In Vivo and In Vitro, Related to Figure 3 (Collision assay) This is an example of Mig-NC collisions in which cells undergo CIL (left) and of Mig-NC expressing E-cadherin forming a stable contact (right). Magenta is nuclear-mCherry. Green is membrane GFP. Frame delay is 5 minutes. Magnification is ×20. (Dispersion assay) Mig-NC explants undergo EMT and disperse (left), while Mig-NC expressing E-cadherin remains in a more compact cluster (right). Magenta is nuclear-mCherry. Green is membrane GFP. Frame delay is 5 minutes. Magnification is ×10. (*Sox10:egfp*) Zebrafish embryos. *sox10:egfp* NC (left) is strongly polarized and produce protrusions directed outward (arrow), while in E-cadherin expressing *sox10:egfp* embryos (right), NC protrusions are not polarized. Channel is GFP. Frame delay is 4 minutes. Magnification is ×50. (Protrusion analysis) Mig-NC (left) are strongly polarized and produce protrusions directed outward (arrow), while in E-cadherin expressing Mig-NC (right) protrusions are not polarized (arrow) and produce protrusions at cell-cell contact sites (arrowhead). Magenta is nuclear-mCherry. Green is membrane GFP. Frame delay is 10 seconds. Magnification is ×60.

Movie S4. p120 Knockdown Rescues Protrusion Formation in E-Cadherin Expressing Migratory NC, Related to Figure 4 (Protrusion analysis) Mig-NC (top left) or Mig-NC +p120 MO (top right) are polarized and produce protrusions directed outwards (arrow), while in E-cadherin expressing Mig-NC, protrusions are not polarized (arrow, bottom left). P120 knockdown restores normal protrusion polarity in E-cadherin expressing NC (arrow, bottom right). Magenta is nuclearmCherry. Green is membrane GFP. Frame delay is 10 seconds. Magnification is ×60.

Movie S5. Repolarization of Protrusions and of Rac1 Activity Precedes Junctional Disassembly during CIL, Related to Figure 5 (Mig-NC cell-cell collision) p120-GFP (green) and lifeactmCherry (magenta) are in left, and Rac1 FRET (heatmap) is in right. Protrusions formation (arrow, left) and Rac1 activation (arrow, right) at the free edge correlate with the disassembly of the p120- GFP positive cell-cell junction (arrowhead, left). Frame delay is 1 minute. Magnification is ×60.

Movie S6. Cell Confinement Inhibits CIL, Related to Figure 5 (Left column) Cell confinement inhibits CIL. This is an example of Mig-NC collisions in unconstrained (top) Fn and of Mig-NC confined in a H-shaped (middle) or disc-shaped micropattern (bottom). Magenta is nuclear-mCherry. Green is membrane GFP. Frame delay is 5 minutes. Magnification is ×60. (Central column) Mig-NC on unconstrained FN; (top) NCadherin-mCherry; (middle) p120-GFP; (bottom) α-catenin-GFP injected NC. (Right column) Mig-NC confined on a disc micropattern, cells forming a stable junction; (top) N-cadherin-mCherry; (middle) p120-GFP; (bottom) α-catenin-GFP injected NC. Frame delay is 3 minutes. Magnification is ×60.

Movie S7. Protrusion Repolarization via Rac1 Is Sufficient to Trigger Cell Separation during CIL, Related to Figure 6 (Photocativation of Rac1 on single cells) PA-Rac promotes protrusion formation in single NC cells. The boxed area was illuminated with 45-second pulses of 514 nm control laser light (left) or with 458 nm wavelength (right). Red is PA-Rac-mCherry Grey is transmitted light. Frame delay is 1 minute. Magnification is ×60. (Photoactivation of dom-neg Rac1 on single cells) DN-PA-Rac promotes protrusion collapse in single NC cells. The boxed area was illuminated with 45-second pulses of 514 nm control laser light (left) or with 458 nm wavelength (right). Red is DN-PA-Rac-mCherry. Grey is transmitted light. Frame delay is 1 minute. Magnification is ×60. (Photoactivation of dom-neg Rac1 on pair of cells) DN-PA-Rac inhibits cell dissociation in Mig-NC doublets. The boxed areas were illuminated with 45-second pulses of 514 nm control laser light (left) or with 458 nm wavelength (right). Red is PA-Rac-mCherry Green is membrane-GFP. Frame delay is 1 minute. Magnification is ×60. (Photoactivation of Rac1 on pair of cells overexpressing E-cadherin) PA-Rac promotes cell dissociation in E-cadherin expressing NC doublets. The boxed areas were illuminated with 45-second pulses of 514 nm control laser light (left) or with 458 nm wavelength (right). Red is PA-Rac-mCherry. Green is E-cadherin-GFP. Frame delay is 1 minute. Magnification is ×60. (Photoactivation of Rac1 on pair of cells overexpressing E-cadherin-GFP) PA-Rac activation by illumination does not affect E-cadherin junctional recruitment. E-cadherin-GFP is injected Mig-NC cells. The boxed areas were illuminated with 45-second pulses of 514 nm control laser light (left) or with 458 nm wavelength (right). Frame delay is 1 minute. Magnification is ×60.

Document S2. Article plus Supplemental Information
