# Supplementary material for: Cadherin Switch during EMT in Neural Crest Cells Leads to Contact Inhibition of Locomotion via Repolarization of Forces
Source: Dev Cell. 2015 Aug 24;34(4):421–34. doi: 10.1016/j.devcel.2015.06.012 (PMC4552721; doi:10.1016/j.devcel.2015.06.012)
Supplement: Document S1. Supplemental Experimental Procedures and Figures S1–S7 [file mmc1.pdf]

Developmental Cell

Supplemental Information

Cadherin Switch during EMT in Neural Crest Cells Leads to Contact Inhibition of Locomotion via Repolarization of Forces

Elena Scarpa, András Szabó, Anne Bibonne, Eric Theveneau, Maddy Parsons, and Roberto Mayor

Supplemental Figures

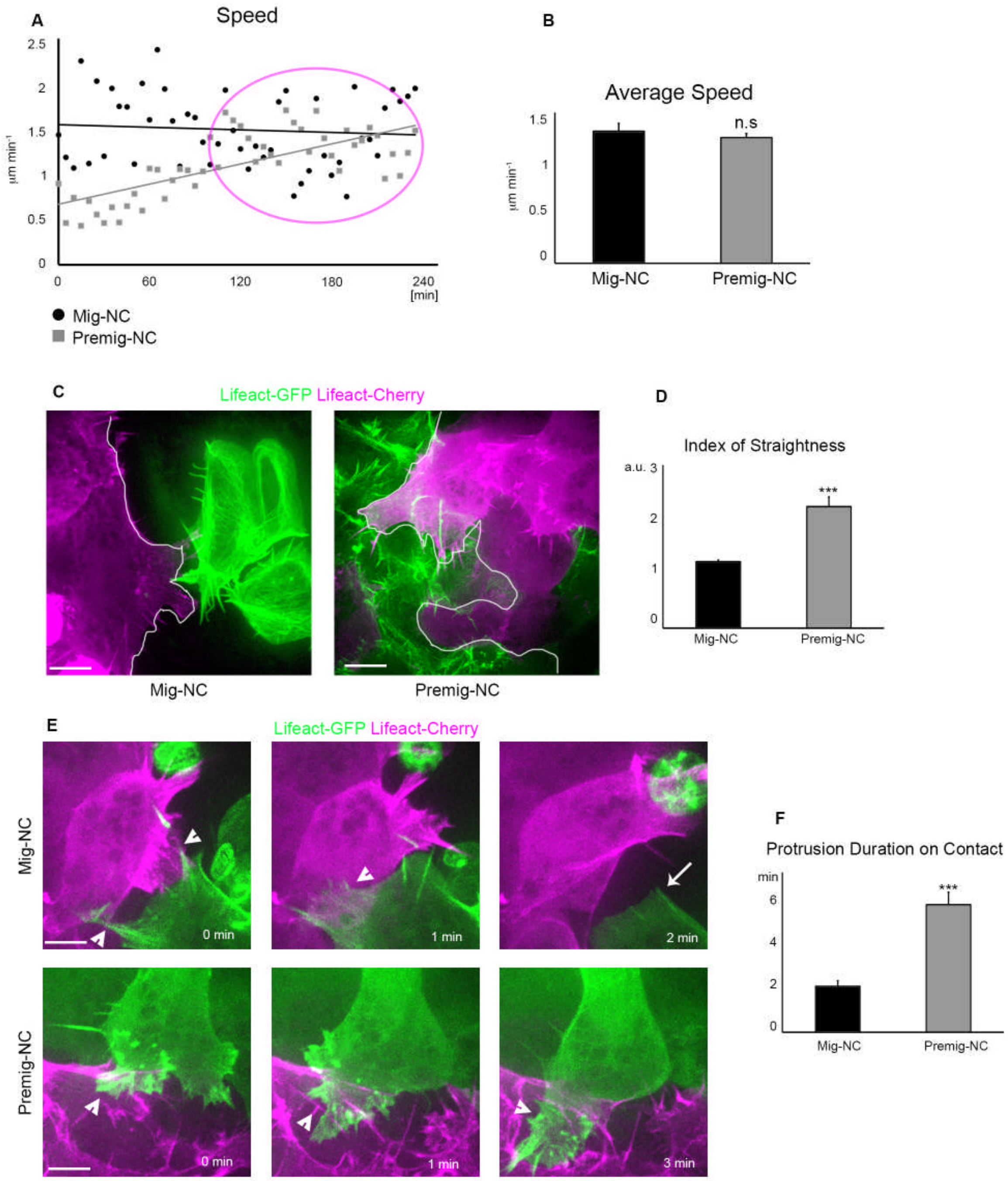

Supplemental Figure S1. **Related to Figure 1.** (A, B) Diagram of speed over time for Mig-NC and Premig-NC. Time 0 corresponds at the time which the NC are plated in vitro. Premig-NC start to migrate slowly, but soon they migrate at the same speed that Mig-NC. In the rest of this work collisions were analysed during timepoints 120-240 (circled in magenta), when the average speed of Mig-NC and Premig-NC was similar. (B) Average speed (Mig-NC n=28 cells, Premig-NC n=39 cells; n.s. not significant). (C) Time lapse stills of an explant overlap assay, outline indicates the boundary between the explants. Scale bar 10 μm. (D) Index of straightness for Mig-NC and Premig-NC (Mig-NC n= 9 explants Premig-NC n=8

explants), *** p<0.001). (E) Time lapse stills of an explant overlap assay, arrowheads: protrusions; arrow: protrusion collapsing. Scale bar 5 µm. (F) Protrusion duration upon contact for Mig-NC and Premig-NC (Mig-NC n= 32, Premig-NC n=23 protrusions) *** p<0.001. All box and whiskers charts: box: median±25th/75th percentile, whiskers: min/max value, bar charts: mean ± s.e.m.

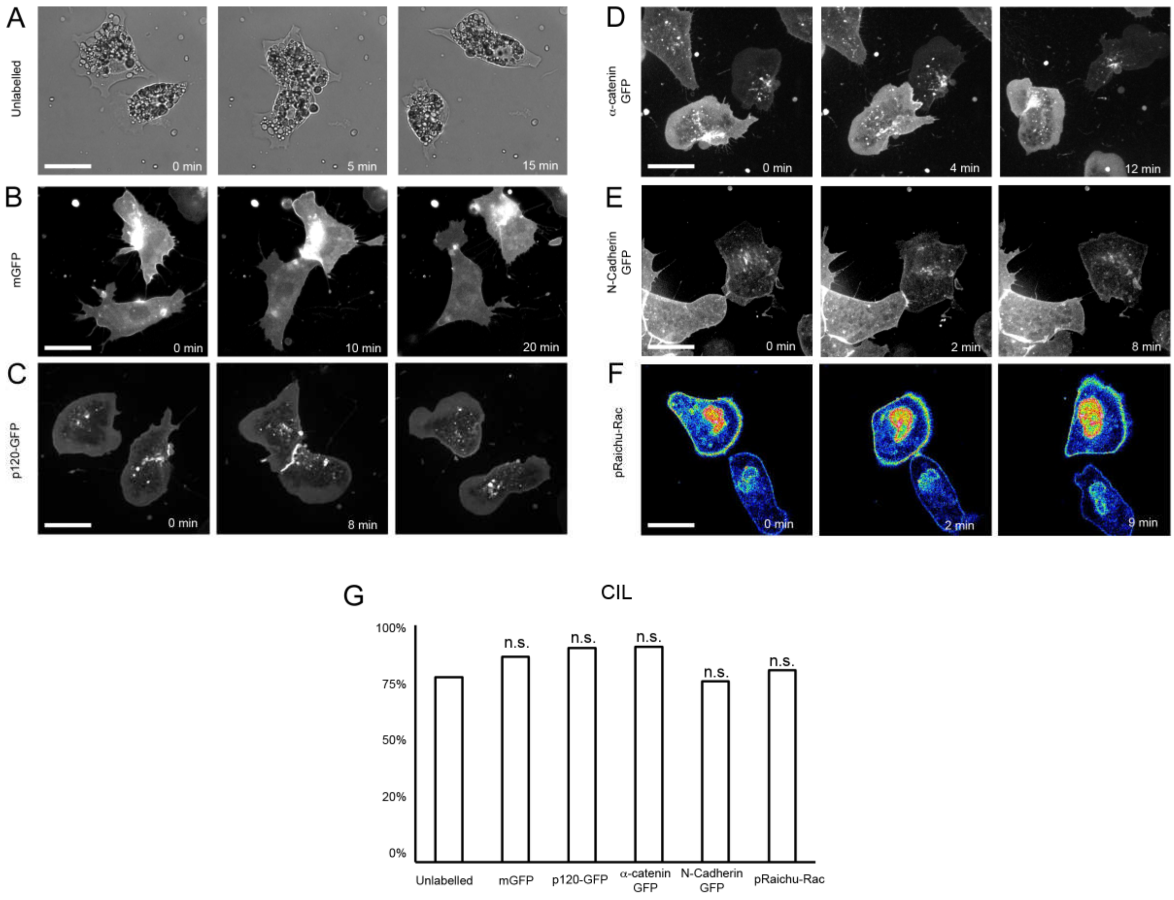

Supplemental Figure S2. **Related to Figure 2.** (A) Time lapse stills for wildtype Mig-NC. (B-F) Time lapse still for NC injected with mRNAs as indicated. Scale bars 20 µm. (G) Percentage of CIL (unlabelled n= 47, mGFP n=68, p120-GFP n=16, α-catenin-GFP n=22, N-Cadherin GFP n=51, p-Raichu-Rac n=22 collisions, n.s. not significant). All box and whiskers charts: box: median±25th/75th percentile, whiskers: min/max value, bar charts: mean ± s.e.m.

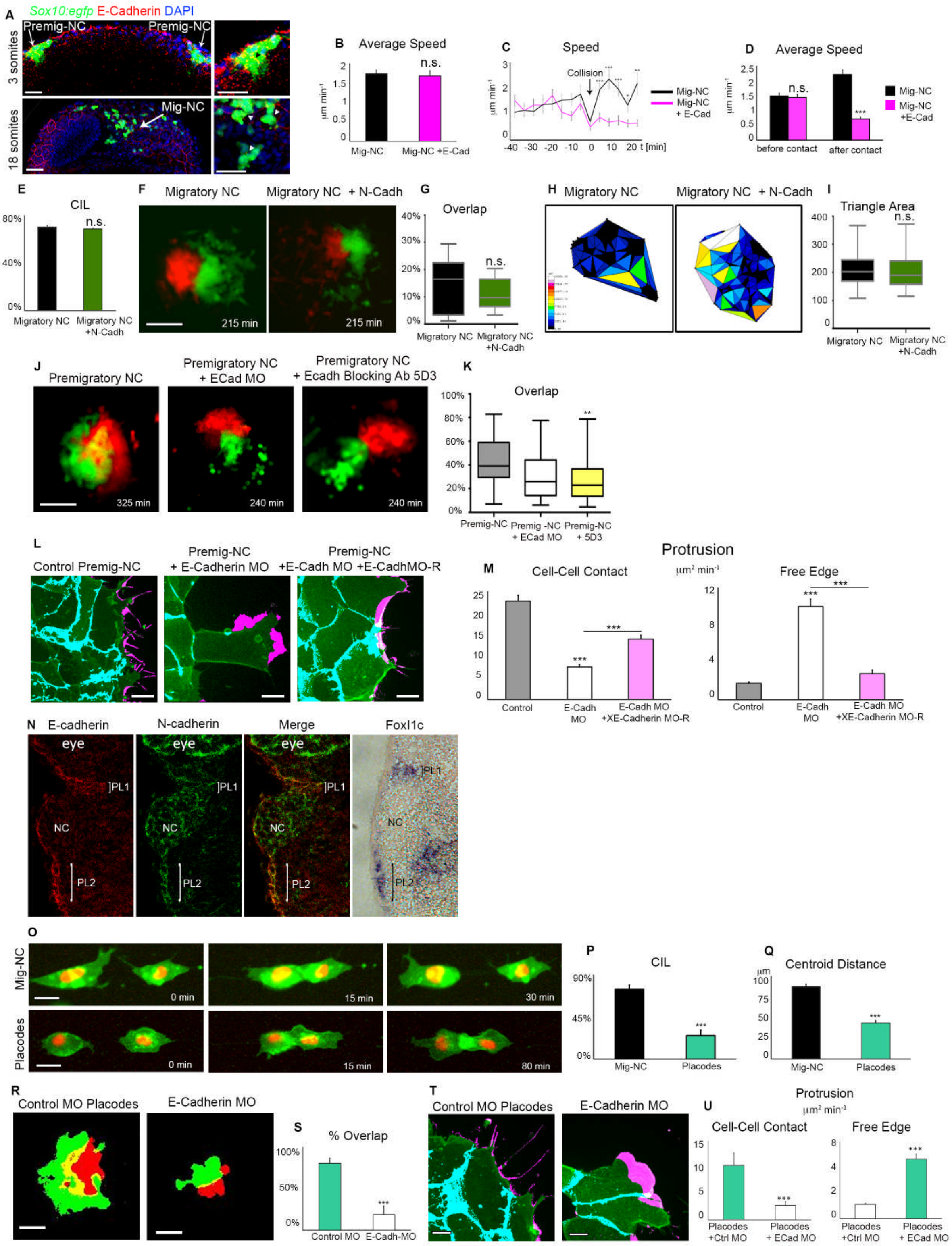

Supplemental Figure S3. **Related to Figure 3.** (A) Expression of endogenous E-Cadherin in *Sox10:egfp* zebrafish embryos at 3 somites (top) and 18 somites (bottom), scale bar 50 µm. (B) Speed of single dissociated cells for Mig-NC and Mig-NC+E-Cadh (n=65 Mig-NC, n= 74 cells Mig-NC+E-Cadh), n.s. not significant. (C) Plot of speed over time for Mig-NC and Mig-NC+E-Cadherin cell-cell collisions. Only collisions in which cells maintained the cell-cell contact were analysed for Mig-NC+E-Cadh. Arrow: timepoint of collision (n= 16 Mig-NC, n=29 collisions Mig-NC+E-Cadherin*p<0.05, **p<0.01, ***p<0.001. (D) Average speed of

single cells before and after contact for Mig-NC and Mig-NC+E-Cadh (n= 16 Mig-NC, n=29 collisions Mig-NC+E-Cadherin)***p<0.001. (E-I) Overexpression of N-cadherin does not affect CIL. (E) Percentage of CIL (n=47 collisions Mig-NC, n=86 collisions Mig-NC+N-Cadh n.s. not significant. (F) Explant overlap assay for Mig-NC or Mig-NC+N-Cadh, scale bar 100 μm. (G) Percentage of overlap between the explants (n= 19 explants Mig-NC, n=18 explants Mig-NC+N-Cadh) n.s. not significant. (H) Dispersion Assay. Colour coded Delaunay triangulation diagrams. (I) Triangle Area at 400 minutes after plating (Mig-NC n=28, Mig-NC+N-Cadh n=27 explants) n.s. not significant. (J) Time lapse stills of explant overlap assay for Premig-NC or Premig-NC+E-Cadh MO or E-Cadh blocking antibody (5D3), scale bar 100 μm. (K) Percentage of overlap between the explants at the time point of maximum superimposition. (Premig-NC n= 33, Premig-NC+E-Cadh n=7 MO, Premig-NC+5D3 n=31 explants) **P<0.01, n.s. not significant. (L) Protrusive activity of Premig-NC+ Control MO, Premig-NC+E-Cadh MO or Premig-NC+E-Cadh MO+E-Cadh mRNA. Still time-lapse photographs of a maximal projection, Free Edge protrusions labelled in magenta, Cell-Cell contact protrusions labelled in cyan (Right), scale bar 10 μm. (M) Quantitation of Protrusion area per minute per cell obtained by subtraction analysis (n= 97 cells Premig-NC+Standard Control MO, n=100 cells Premig-NC+E-Cadh MO, n=156 Premig-NC+E-Cadh MO+E-Cadh mRNA *** P<0.001. (N) Immunostaining for E- and N-Cadherin on *Xenopus* cryosection, in situ hybridization for placodal marker FoxI1c on serial section (right) Brackets: Placodes. (O) Single cell collisions of Mig-NC and Placodal (PL) cells. Scale bar 20 μm. (P) Percentage of CIL for Mig-NC and PL (n=65 Mig-NC, n=50 collisions PL) *** alpha > 0.1%(Q) Distance between nuclei 30 minutes after contact for Mig-NC and PL (n=65 Mig-NC, n=50 collisions PL), *** p<0.001.(R) Time lapse stills of explant overlap assay for PL+Control MO or PL+E-Cadh MO, scale bar 60 μm. (S) Percentage of overlap between the explants at the time point of maximum superimposition.(PL Control-MO n= 11, PL+E-Cadh MO n=5 explants) *** p<0.001. (T) Protrusive activity of PL+ Control MO, PL+E-Cadh MO. Still time-lapse photographs of a maximal projection, Free Edge protrusions labelled in magenta, Cell-Cell contact protrusions labelled in cyan (Right), scale bar 10 μm. (U) Quantitation of Protrusion area per minute per cell obtained by subtraction analysis (PL Control-MO n= 50, PL+E-Cadh MO n=51 cells, *** p<0.001. All box and whiskers charts: box: median±25th/75th percentile, whiskers: min/max value, bar charts: mean±s.e.m.

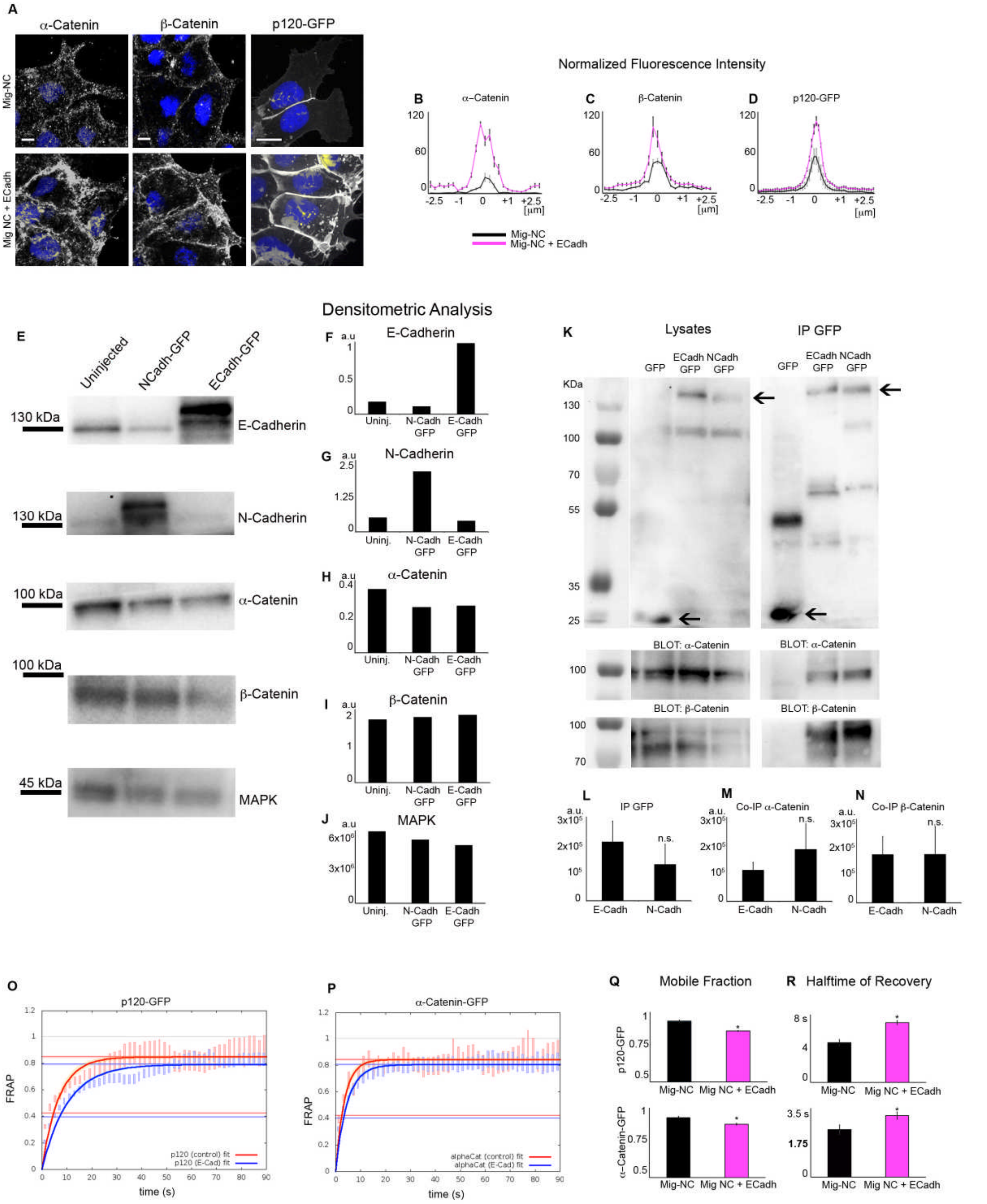

Supplemental Figure S4. **Related to Figure 3.** (A) Immunostaining for α-catenin and β-catenin, fluorescence image for p120-GFP in Mig-NC and Premig-NC, scale bars 10 µm, nuclear staining: DAPI. (B,C, D) Fluorescence intensity across cell-cell junctions normalised to fluorescence in adjacent cell cytoplasm (α-catenin: Mig-NC n=50; Mig-NC+E-Cadh n=50, 2 independent experiments, β-catenin: Mig-NC n=50; Mig-NC+E-Cadh n=50; p120-GFP: Mig-NC n=30; Mig-NC+E-Cadh n=30,3 independent experiments). (E) Western blot (WB) of stage 19 embryo lysates. Antibody blotting as illustrated. (F-J) Densitometric analysis of the WB shown in (E), with (F-I) representing ratio between the antibody band intensity and the pixel intensity for MAPK (J). (K) Immunoprecipitation (IP) of GFP, E-cadh-GFP, N-Cadh-GFP from Stage 19 embryo lysates. A WB of lysates is shown in left panel. Pull down of GFP is

shown in right panel. Coimmunoprecipitation of α- and β-Catenin and their corresponding lysates is shown in the bottom panels. (L-N) Pixel volumetric intensity of GFP IP (L) and corresponding co-immunoprecipitates for α-catenin (M) and β-Catenin (N) (n=5 independent experiments, error bars: s.e.m, n.s. not significant. (O, P). FRAP single exponential fits for recovery curves for p120-GFP (O) and α-Catenin-GFP (P), vertical bars: s.d of FRAP data. Shades around fitting curves: 95% confidence intervals (c.i). (Q, R) Fitting FRAP parameters for p120-GFP (top) and α-catenin-GFP (bottom) in Mig-NC and Mig-NC+E-Cadh. Mobile fraction in (Q), Halftime of recovery in (R) (n=18 FRAP regions for Mig-NC, n=24 FRAP regions for Mig-NC+ECadherin for p120-GFP; n=29 FRAP regions for Mig-NC, n=36 FRAP regions for Mig-NC+ECadherin for α-catenin-GFP FRAP, 3 independent experiments, error bars: 95% c.i., *P<0.05). Bar charts: mean±s.e.m.

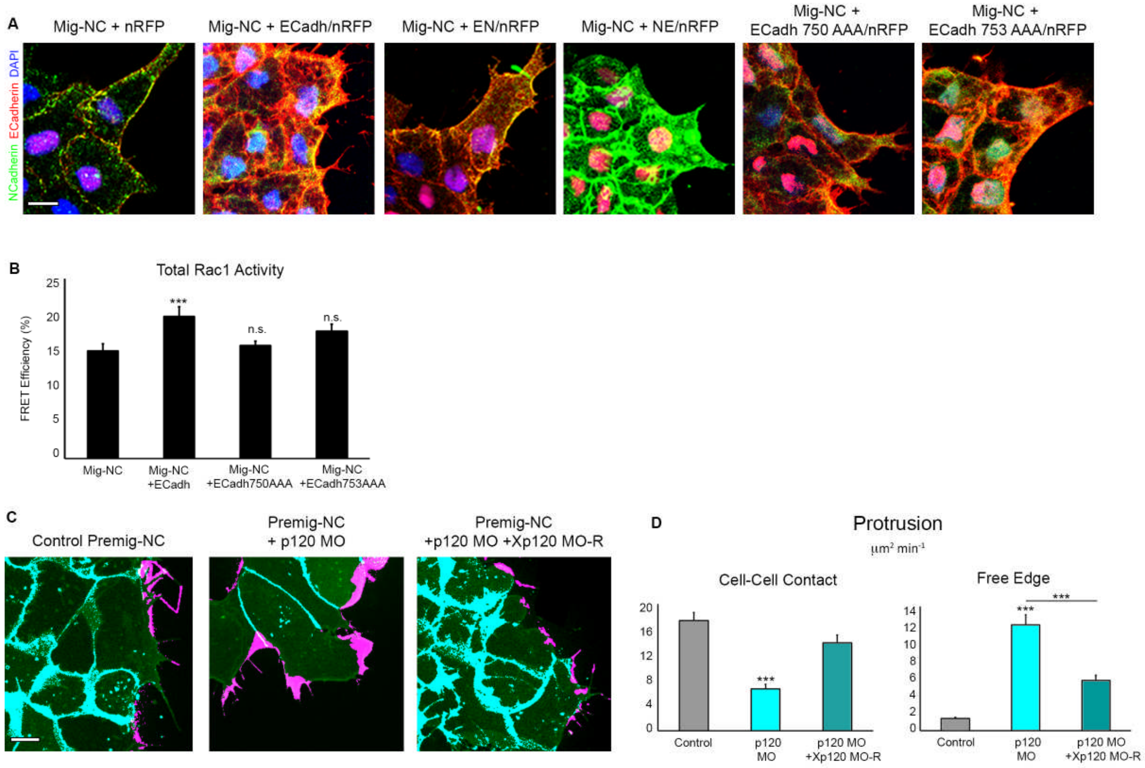

Supplemental Figure S5. **Related to Figure 4.** (A) Double immunostaining for E- and N-Cadherin in Mig-NC, Mig-NC+E-Cadh, Mig-NC+E/N, Mig-NC+N/E, Mig-NC+E-Cadh750AAA, Mig-NC+E-Cadh753AAA, scale bar 10 µm. Both antibodies are specific for the EC domain of each cadherin, therefore distinguishing the expression of the swapped CYTO domains mutants. (B) Rac1 FRET efficiency measured by acceptor photobleaching for Mig-NC, Mig-NC+E-Cadh and the p120-uncoupled mutants E-Cadh750AAA and E-Cadh753AAA (n=16 Mig-NC, n=22 Mig-NC+E-Cadh, n=15 Mig-NC+E-Cadh750AAA, n=12 Mig-NC+E-Cadh753AAA * P<0.05, *** P<0.001. (C) Protrusive activity of Premig-NC+ Control MO, Premig-NC+p120 MO or Premig-NC+p120 MO+Xp120 mRNA. Still time-lapse photographs of a maximal projection, Free Edge protrusions labelled in magenta, Cell-Cell contact protrusions labelled in cyan (Right), scale bar 10 µm. (D) Quantitation of Protrusion area per minute per cell obtained by subtraction analysis (n= 104 cells Premig-NC+Control MO, n=95 cells Premig-NC+p120 MO, n=103 Premig-NC+p120 MO+Xp120 mRNA*** P<0.001. Charts: mean ± s.e.m.

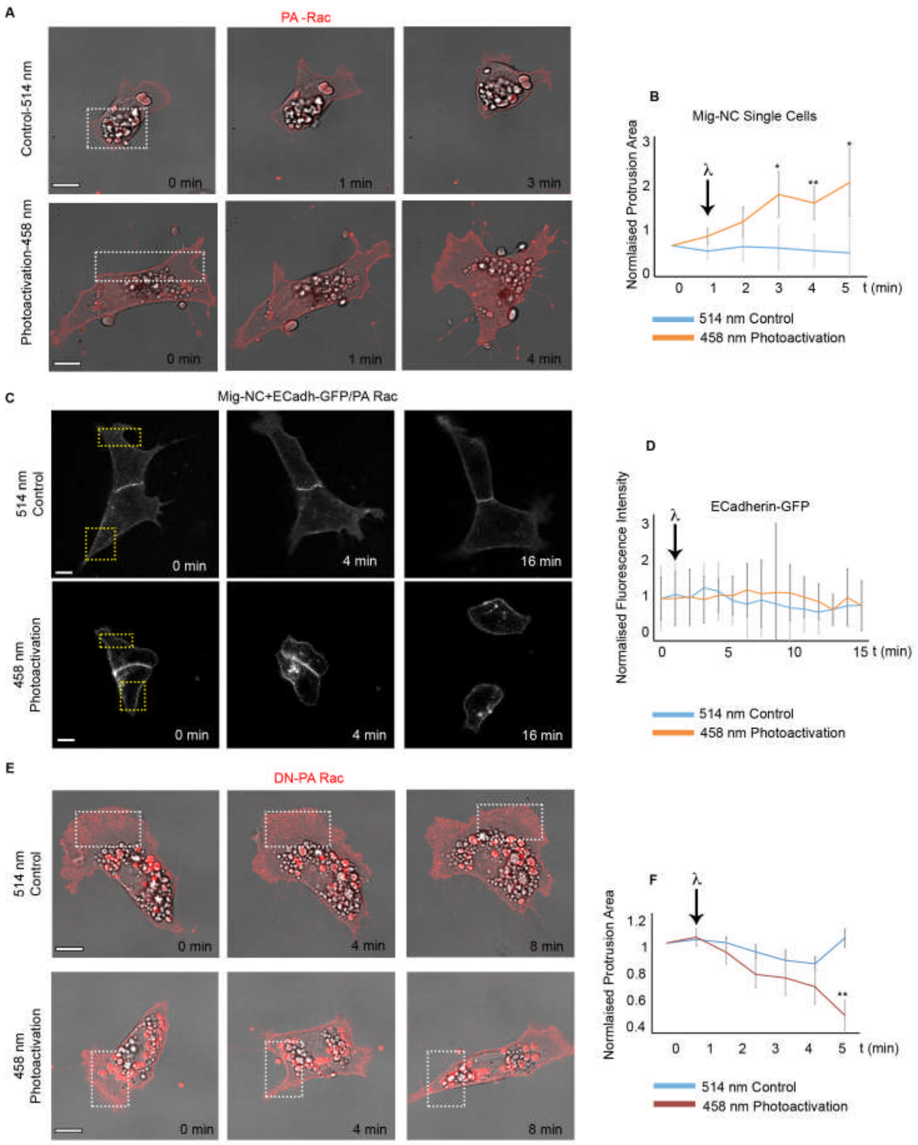

Supplemental Figure S6. **Related to Figure 6.** (A) Photograms from a photoactivation experiment. Mig-NC expressing PA-Rac-Cherry. Illumination of the area in the box (white dashed lines) with 514 nm control wavelength (top) does not trigger cell protrusion; illumination of the area in the box (white dashed lines) with 458 nm wavelength results in formation of a new protrusion(bottom), scale bar 10 µm. (B) Protrusion area over time. Illumination occurs at t=1 min (arrow) (514 nm n=5 cells, 458 nm n=6 cells, * P<0.05 **P<0.01). (C) Time lapse stills from a photoactivation experiment. Mig-NC expressing E-Cadh-GFP and PA-Rac Cherry. Illumination of the area in the box (yellow dashed lines) with 514 (top)or 458 nm (bottom) wavelength does not alter E-Cadh-GFP localization, scale bar 10 µm. (D) Normalised E-Cadh-GFP junctional intensity over time. Illumination occurs at t=1 min (arrow) (514 nm n=6 cells, 458 nm n=6 cells). (E) Photograms from a DN-Rac photoactivation experiment. Mig-NC expressing DN-PA-Rac-Cherry. Illumination of the area in the box (white dashed lines) with 514 nm control wavelength (top) does not trigger protrusion collapse; illumination of the area in the box (white dashed lines) with 458 nm wavelength results in collapse of the protrusion in the illumination area(bottom), scale bar 10 µm. (F) Protrusion area over time. Illumination occurs at t=1 min (arrow) (514 nm n=5 cells, 458 nm n=5 cells, **P<0.01).Charts: mean ±s. e.m.

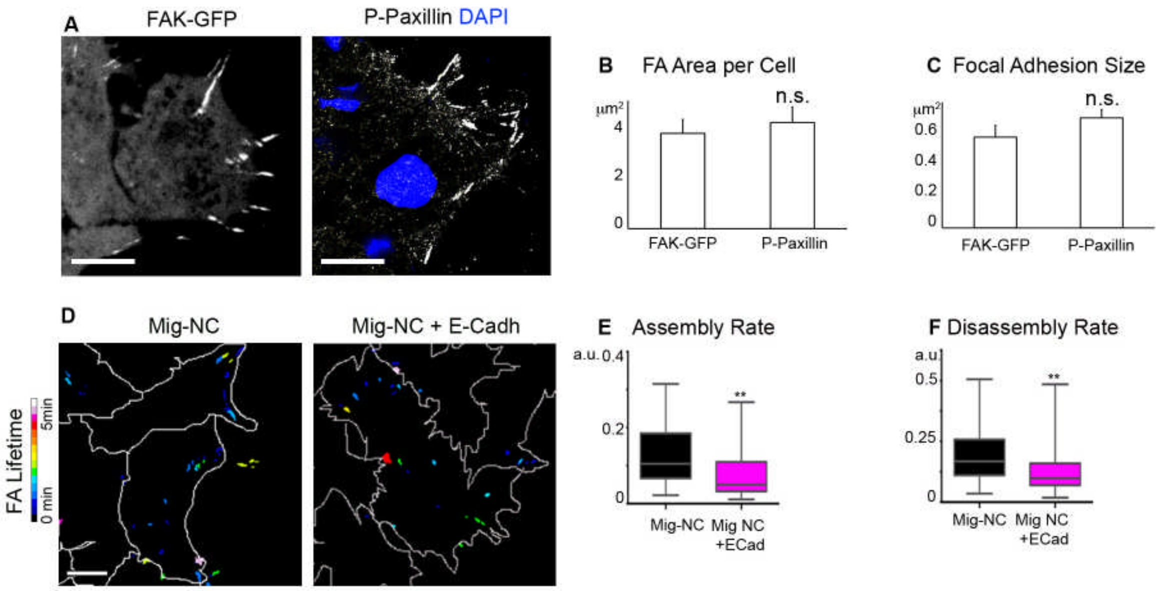

Supplemental Figure S7. **Related to Figure 7.** (A-C) Comparison between endogenous FA and FA observed by FAK-GFP accumulation. (A) Left: Fluorescence image of FAK-GFP, note GFP accumulation in the arears expected for FA; right: FA detected by P-Paxillin staining. No difference in the FA per cell (B) or FA size (C) was observed between P-Paxillin staining and FAK-GFP. (D) Heatmap of focal adhesion (FA) lifetime in Mig-NC and Mig-NC+E-Cadh, scale bar 10 μm. (E) Assembly Rate of FA in Mig-NC and Mig-NC+E-Cadh (Mig-NC n=58, Mig-NC+E-Cadh n=31 **P<0.01 Mann-Whitney test). (F) Disassembly Rate of FA in Mig-NC and Mig-NC+E-Cadh (Mig-NC n=57 , Mig-NC+E-Cadh n=40 **P<0.01 . All box and whiskers charts: box: median±25th/75th percentile, whiskers: min/max value, bar charts: mean±s.e.m.

Legends for Supplemental Movies

Supplemental Movie S1. Related to Figure 1. CIL is a developmentally regulated property of Neural Crest Cells. (**Collision assay**). An example of Mig-NC collisions in which cells undergo CIL (left) and of Premig-NC, cells forming a stable contact (right). Magenta: nuclear-mCherry. Green: membrane GFP. Frame delay: 5 minutes. Magnification 20X. (**Overlap assay**). An example of Mig-NC explant overlap assay in which explants undergo CIL and do not overlap (left) and of Premig-NC, in which explants overlap and cells intermingle (right). Magenta: Rhodamine Dextran. Green: Fluorescein-Dextran. Frame delay: 5 minutes. Magnification 10X. (**Dispersion assay**). Mig-NC explants undergo EMT and disperse (left) while Premig-NC do not disperse (right). Magenta: nuclear-mCherry. Green: membrane GFP. Frame delay: 5 minutes. Magnification 10X. (**Protrusion analysis**). Mig-NC are strongly polarised and produce protrusions directed outwards (arrow, left panel) while Premig-NC protrusions are not polarised (arrow, right) and produce protrusions at cell cell contact sites (arrowhead). Magenta: nuclear-mCherry. Green: membrane GFP. Frame delay: 10 seconds. Magnification 60X. (**Lifeact-GFP**). Mig-NC are strongly polarised and produce actin-rich protrusions directed outwards (arrow, left panel) while Premig-NC protrusions are not polarised(arrow, right) and produce actin-rich protrusions at cell cell contacts (arrowhead). Frame delay: 10 seconds. Magnification 60X.

Supplemental Movie S2. Related to Figure 2. **Different dynamics of junction disassembly in Migratory and Premigratory Neural Crest Cells.** An example of Mig-NC collisions in which cells undergo CIL (**left**) and of Premig-NC, cells forming a stable junction (**right**). (**Top**) p120-GFP (green). (**Bottom**) □-catenin-GFP (green). Magenta: nuclear-mCherry. Frame delay: 20 seconds. Magnification 60X.

Supplemental Movie S3. Related to Figure 3. **E-Cadherin suppresses CIL *in vitro* and *in vivo*.** (**Collision assay**). An example of Mig-NC collisions in which cells undergo CIL (left) and of Mig-NC expressing E-Cadherin forming a stable contact (right). Magenta: nuclear-mCherry. Green: membrane GFP. Frame delay: 5 minutes. Magnification 20X. (**Dispersion assay**). Mig-NC explants undergo EMT and disperse (left) while Mig-NC expressing E-Cadherin remain in a more compact cluster (right). Magenta: nuclear-mCherry. Green: membrane GFP. Frame delay: 5 minutes. Magnification 10X. (***Sox10:egfp***). Zebrafish embryos. *sox10:egfp* neural crest (left) are strongly polarised and produce protrusions directed outwards (arrow) while in E-Cadherin expressing *sox10:egfp* embryos (right) neural crest protrusions are not polarised. Channel: GFP. Frame delay: 4 minutes. Magnification 50X. (**Protrusion analysis**). Mig-NC (left) are strongly polarised and produce protrusions directed outwards (arrow) while in E-Cadherin expressing Mig-NC (right) protrusions are not polarised (arrow) and produce protrusions at cell-cell contact sites (arrowhead). Magenta: nuclear-mCherry. Green: membrane GFP. Frame delay: 10 seconds. Magnification 60X.

Supplemental Movie S4. Related to Figure 4. P120 knockdown rescues protrusion formation in E-Cadherin expressing Migratory Neural Crest. Protrusion Analysis. Mig-NC (**top left**) or Mig-NC +p120 MO (**top right**) are polarised and produce protrusions directed outwards (arrow) while in E-Cadherin expressing Mig-NC protrusions are not polarised (arrow, **bottom left**). P120 knockdown restores normal protrusion polarity in E-Cadherin expressing NC (arrow, **bottom right**). Magenta: nuclear-mCherry. Green: membrane GFP. Frame delay: 10 seconds. Magnification 60X.

Supplemental Movie S5. Related to Figure 5. Repolarization of protrusions and of Rac1 activity precedes junctional disassembly during CIL. Mig-NC cell-cell collision.

p120-GFP (green) and lifeact-mCherry (magenta) in **left panel**, Rac1 FRET (heatmap) in **right panel**. Protrusions formation (arrow, left) and Rac1 activation (arrow, right) at the free edge correlate with the disassembly of the p120-GFP positive cell-cell junction (arrowhead, left). Frame delay: 1 minute. Magnification 60X.

Supplemental Movie S6. Related to Figure 5. **Cell confinement inhibits CIL**.(**Left column**) Cell confinement inhibits CIL. An example of Mig-NC collisions in unconstrained (**top**) Fn, and of Mig-NC confined in a H shaped (**middle**) or disc shaped micropattern (**bottom**). Magenta: nuclear-mCherry. Green: membrane GFP. Frame delay: 5 minutes. Magnification 60X. (**Central column**) Mig-NC on unconstrained FN; (**top**) N-Cadherin-mCherry; (**middle**) p120-GFP; (**bottom**) □-catenin-GFP injected NC. (**Right column**). Mig-NC confined on a disc micropattern, cells forming a stable junction; (**top**) N-Cadherin-mCherry; (**middle**) p120-GFP; (**bottom**) □-catenin-GFP injected NC. Frame delay: 3 minutes. Magnification 60X.

Supplemental Movie S7. Related to Figure 6. **Protrusion repolarization via Rac1 is sufficient to trigger cell separation during CIL**.(**Photoactivation of Rac1 on single cells**) PA-Rac promotes protrusion formation in single NC cells. The boxed area was illuminated with 45 seconds pulses of 514 nm control laser light (left) or with 458 nm wavelength (right). Red: PA-Rac-mCherry Grey: transmitted light. Frame delay: 1 minute. Magnification 60X. (**Photoactivation of dom-neg Rac1 on single cells**) DN-PA-Rac promotes protrusion collapse in single NC cells. The boxed area was illuminated with 45 seconds pulses of 514 nm control laser light (left) or with 458 nm wavelength (right). Red: DN-PA-Rac-mCherry Grey: transmitted light. Frame delay: 1 minute. Magnification 60X. (**Photoactivation of dom-neg Rac1 on pair of cells**) DN-PA-Rac inhibits cell dissociation in Mig-NC doublets. The boxed areas were illuminated with 45 seconds pulses of 514 nm control laser light (left) or with 458 nm wavelength (right). Red: PA-Rac-mCherry Green: membrane-GFP. Frame delay: 1 minute. Magnification 60X. (**Photoactivation of Rac1 on pair of cells overexpressing E-cadherin**) PA-Rac promotes cell dissociation in E-Cadherin expressing NC doublets. The boxed areas were illuminated with 45 seconds pulses of 514 nm control laser light (left) or with 458 nm wavelength (right). Red: PA-Rac-mCherry Green: E-Cadherin-GFP. Frame delay: 1 minute. Magnification 60X. (**Photoactivation of Rac1 on pair of cells overexpressing E-cadherin-GFP**) PA-Rac activation by illumination does not affect E-cadherin junctional recruitment. E-Cadherin-GFP injected Mig-NC cells. The boxed areas were illuminated with 45 seconds pulses of 514 nm control laser light (left) or with 458 nm wavelength (right). Frame delay: 1 minute. Magnification 60X.

Supplemental Experimental Procedures

Microinjection. *Xenopus laevis* embryos were injected at the 8-cell stage in the dorsal and ventral animal blastomeres as previously described (Carmona-Fontaine et al., 2008). mRNA encoding the following proteins was injected: membrane targeted GFP (300 pg), nuclear mCherry (300 pg), lifeact-GFP (400pg), lifeact-mCherry (500 pg), N-cadherin-mCherry (1 ng), p120–GFP (500 pg), α-catenin–GFP (500 pg), FAK-GFP (400 pg), pRaichu-Rac1(Itoh et al., 2002) (1 ng, FRET), Vinculin-TS FRET plasmid DNA(Grashoff et al., 2010) (800 pg), PA-Rac-mCherry (500 pg), DN-PA-Rac-mCherry (500 pg), XE-cadherin and XE-Cadherin-GFP (400-800 pg).

Embryology. For *in vitro* experiments, explants were dissected either at stage 15 or at stage 19 (Nieuwkoop and Faber) and plated on a fibronectin-coated dish as described in (Theveneau et al., 2010) or on a fibronectin micropatterned coverslip (CYTOO). Briefly, sealable plastic Petri dishes or glass bottomed dishes were incubated with a 10 or 100 μg ml^{-1} fibronectin solution respectively for 1 h at 37° and for 30 min in PBS containing 0.1% albumin. Explants were plated on coated dishes containing modified Danilchick's medium(Theveneau et al., 2010) and left to adhere for 20 min before the beginning of the time-lapse recordings. Transgenic *sox10:egfp* (Carney et al., 2006) embryos were obtained by crossing heterozygous adults. *sox10:egfp* was used to analyze NC migration in vivo (Carney et al., 2006). Embryos were processed as previously described (Matthews et al., 2008). Each embryo was staged according to the number of somites and only embryos with equal numbers of somites were compared. The embryos were dechorionated, inserted into a drop of 0.20% agarose in embryo medium (Westerfield, 2000) and mounted in a custom-built chamber for imaging.

Collision analysis and invasion assays. For single cell collision assays, NC cells were briefly dissociated in Ca^{2+}/Mg^{2+} free Danilchick medium and plated on fibronectin coated dishes. The CIL response was assessed by counting the percentage of collisions in which cells have separated 30 minutes after contact initiation. The distance between the cell nuclei was measured 30 min after cells have separated (Scarpa et al., 2013; Theveneau et al., 2013). Invasion assays were performed as previously described (Carmona-Fontaine et al., 2008; Theveneau et al., 2010). Briefly, two explants labelled with different fluorescent markers were placed at about 50 μm distance from one another and left to migrate. Time lapse images were acquired every 5 minutes for 10 hours. The fluorescence micrographs were then thresholded and the area of maximum overlap between the green and red channels was measured and normalized to the area of the smaller of the two explants.

Immunostaining and antibodies, antisense MOs. Immunostaining of *Xenopus* NC was performed as previously described (Moore et al., 2013). Briefly, they were cultured on fibronectin-coated glass coverslips for 4-6 hour. Cells were then fixed in 3.7% formaldehyde for 30 minutes, washed with PBS and permeabilised with PBS 0.1% Triton-X-100, blocked for 30 minutes in PBS 5%BSA-3% serum, before incubation of the primary and secondary antibodies in blocking buffer. The following antibodies were used: N-cadherin (rat IgG, clone MNCD2, DSHB; 1 in 50), E-cadherin (mouse IgG, clone 5D3, DSHB, 1 in 200); α-Catenin (Rabbit IgG Abcam AB51032, 1 in 50), β-Catenin (Rabbit IgG Sigma, 1 in 200)(Kuriyama et al., 2014). E-cadherin MO and p120 MO were purchased from Gene Tools and used as previously described (Ciesiolka et al., 2004; Nandadasa et al., 2009). Secondary antibodies: donkey anti-mouse–Alexa 555 (Invitrogen, A31570; 1 in 500), goat anti-rat–Alexa488 (LifeTechnologies, 1 in 500), donkey anti-rabbit–Alexa488 (Sigma, 1 in 500). DAPI was used to stain nuclei.

Preparation of embryo lysates and immunoprecipitation. Embryos were lysed in Lysis Buffer (100 mM NaCl, 50 mM Tris-HCl, 1% Triton-X-100) supplemented with the antipain, leupeptin, pepstatin and PMSF (Sigma) at 10 μg/mL each. Immunoprecipitation was performed as described in (Gai et al., 2011). Briefly, embryos were incubated in Lysis Buffer

for 10 minutes on ice, vortexed quickly and centrifuged for 10 minutes at 4 °C at 13000 rpm. The cytoplasmic fraction was recovered. Protein concentration was then determined by using a BCA Protein Assay Kit (Novagen) using a spectrophotometre. For total lysate samples, 50 μg of protein were diluted in sample buffer and denatured for 5 minutes at 95°C. For immunoprecipitation, supernatants were diluted to 6 mg/ml in 250 μl and incubated at 4°C for 3 hours 30 minutes with 10 μl of a 50% slurry of GFP-TRAP beads (Chromotek). Beads were centrifuged for 2 minutes at 2000 rpm at 4°C and washed three times in Lysis Buffer. Bound material was eluted by adding NuPAGE LDS sample buffer and denaturing the samples for 5 minutes at 95°C. Samples were analysed by SDS-PAGE.

Generation of X-ECadherin mutants. cDNA fragments were amplified by PCR using the following primers. For E/N, E-Cad-EC domain Fw 5'-GTTTTGATGTTTGTTGTATGGG TACGAAGAAAGAAAGTGGTA-3'. Rev 5'-CTCCTTGTCACGACGCTTCATAAATAGTAAGAGCA ACAACAG-3'. N-Cad Cytoplasmic domain Fw 5'-GTTTTGATGTTTGTTGTATGGGTACGAAGAAA GAAAGTGGTA-3'

5-CAATAACTCGAGTCAGTCGTCGCTCCCTCCATA-3'. For N/E N-Cad-EC domain Fw 5'-CAATAACTCGAGATGTGCGGGAAAGAGCCCTTC-3' Rev 5'-TACCACTTTCT TTCTTCGTACCCATACAACAAACATCAAAAC-3' E-cad-Cytoplasmic domain Fw 5'-CTGTTGTTGC TCTTACTATTTATGAAGCGTCGTGACAAGGAG-3' Rev 5'-CTACCGTCTAGATTAATCCTCA TCACCTCCATACAT-3'. Fragments was used in a second PCR reaction round to produce a chimaeric amplicon, which was digested and subcloned into pCS2+ using ClaI-XhoI for E/N and XhoI-XbaI for N/E.

Traction forces. The preparation of the polyacrylamide hydrogels containing fluorescent beads was adapted from previously published protocols (Dembo and Wang, 1999; Wang et al., 2002). The concentrations of Acrylamide and Bis-Acrylamide were adjusted to obtain a Young's modulus of 600 Pa. Gels were crosslinked with covalently bound fibronectin to allow attachment of NC cells. Cells were then imaged on an inverted microscope with a ×32 lens and micrographs of the uppermost layer of the gel and of the cells were taken at 5-min intervals. Photographs of the gels in a relaxed state were taken after removing the cells by trypsinization. Traction force measurements were performed as previously described (Lin et al., 2010). Free edge and cell-contact measurement were performed using a custom-built ImageJ plugin. Briefly, a series of selections were made around each explant perimeter or in its centre, in which tractions were averaged and normalized to the overall traction in the total explant area. NC cells were injected with membrane Cherry and the fluorescence signal was used as a reference to locate the cells.

Photoactivation. Photoactivation of PA-Rac and DN-PA-Rac was performed as previously described (Wang et al., 2010; Wu et al., 2009). Briefly, cells were imaged under a Zeiss 710 LSM confocal microscope. FRAP mode was used to image GFP/Cherry and transmitted light. PA was performed using either the 514 nm or the 458 nm laser line at 10% power. A region of interest covering about one third of the cell was selected and illuminated for 45 seconds. Images were acquired every minute for 20-25 minutes.

Statistical analysis. Comparison of percentages was performed using contingency tables as described previously (Carmona-Fontaine et al., 2008).Two data sets were considered significantly different (null hypothesis rejected) if $T>3.841$ ($\alpha = 0.05$,*), $T>6.635$ ($\alpha = 0.01$,**) or $T>10.83$ ($\alpha = 0.001$,***). Normality of data sets was tested using Kolmogorov–Smirnov's test, d'Agostino and Pearson's test using Prism6 (GraphPad). A data set was considered normal if found as normal by the two tests. Data sets following a normal distribution were compared with Student's *t*-test (two-tailed, unequal variances) in Excel or a one-way analysis of variance (ANOVA) with a Dunnett's multiple comparisons post-test in Prism6 (GraphPad). Data sets that did not follow a normal distribution were compared using Mann–Whitney's test or a non-parametric ANOVA (Kruskal–Wallis with Dunn's multiple comparisons post-test) using Prism6 (GraphPad). Cross-comparisons were performed only if the overall P value of the ANOVA was <0.05.

Analysis of Focal Adhesions Dynamics. Focal adhesion assembly and disassembly in FAK-GFP expressing neural crest cells were analysed using the online tool described by (Berginski and Gomez, 2013).

Supplementary References

Berginski, M.E., and Gomez, S.M. (2013). The Focal Adhesion Analysis Server: a web tool for analyzing focal adhesion dynamics. F1000Research *2*, 68.

Carmona-Fontaine, C., Matthews, H.K., Kuriyama, S., Moreno, M., Dunn, G.A., Parsons, M., Stern, C.D., and Mayor, R. (2008). Contact inhibition of locomotion in vivo controls neural crest directional migration. Nature *456*, 957-961.

Carney, T.J., Dutton, K.A., Greenhill, E., Delfino-Machin, M., Dufourcq, P., Blader, P., and Kelsh, R.N. (2006). A direct role for Sox10 in specification of neural crest-derived sensory neurons. Development *133*, 4619-4630.

Ciesiolka, M., Delvaeye, M., Van Imschoot, G., Verschuere, V., McCrea, P., van Roy, F., and Vleminckx, K. (2004). p120 catenin is required for morphogenetic movements involved in the formation of the eyes and the craniofacial skeleton in Xenopus. Journal of cell science *117*, 4325-4339.

Dembo, M., and Wang, Y.L. (1999). Stresses at the cell-to-substrate interface during locomotion of fibroblasts. Biophysical journal *76*, 2307-2316.

Gai, M., Camera, P., Dema, A., Bianchi, F., Berto, G., Scarpa, E., Germena, G., and Di Cunto, F. (2011). Citron kinase controls abscission through RhoA and anillin. Molecular biology of the cell *22*, 3768-3778.

Grashoff, C., Hoffman, B.D., Brenner, M.D., Zhou, R., Parsons, M., Yang, M.T., McLean, M.A., Sligar, S.G., Chen, C.S., Ha, T.*, et al.* (2010). Measuring mechanical tension across vinculin reveals regulation of focal adhesion dynamics. Nature *466*, 263-266.

Itoh, R.E., Kurokawa, K., Ohba, Y., Yoshizaki, H., Mochizuki, N., and Matsuda, M. (2002). Activation of rac and cdc42 video imaged by fluorescent resonance energy transfer-based single-molecule probes in the membrane of living cells. Molecular and cellular biology *22*, 6582-6591.

Kuriyama, S., Theveneau, E., Benedetto, A., Parsons, M., Tanaka, M., Charras, G., Kabla, A., and Mayor, R. (2014). In vivo collective cell migration requires an LPAR2-dependent increase in tissue fluidity. The Journal of cell biology *206*, 113-127.

Lin, Y.C., Tambe, D.T., Park, C.Y., Wasserman, M.R., Trepat, X., Krishnan, R., Lenormand, G., Fredberg, J.J., and Butler, J.P. (2010). Mechanosensing of substrate thickness. Physical review E, Statistical, nonlinear, and soft matter physics *82*, 041918.

Matthews, H.K., Marchant, L., Carmona-Fontaine, C., Kuriyama, S., Larrain, J., Holt, M.R., Parsons, M., and Mayor, R. (2008). Directional migration of neural crest cells in vivo is regulated by Syndecan-4/Rac1 and non-canonical Wnt signaling/RhoA. Development *135*, 1771-1780.

Moore, R., Theveneau, E., Pozzi, S., Alexandre, P., Richardson, J., Merks, A., Parsons, M., Kashef, J., Linker, C., and Mayor, R. (2013). Par3 controls neural crest migration by promoting microtubule catastrophe during contact inhibition of locomotion. Development *140*, 4763-4775.

Nandadasa, S., Tao, Q., Menon, N.R., Heasman, J., and Wylie, C. (2009). N- and E-cadherins in Xenopus are specifically required in the neural and non-neural ectoderm, respectively, for F-actin assembly and morphogenetic movements. Development *136*, 1327-1338.

Scarpa, E., Roycroft, A., Theveneau, E., Terriac, E., Piel, M., and Mayor, R. (2013). A novel method to study contact inhibition of locomotion using micropatterned substrates. Biology open *2*, 901-906.

Theveneau, E., Marchant, L., Kuriyama, S., Gull, M., Moepps, B., Parsons, M., and Mayor, R. (2010). Collective chemotaxis requires contact-dependent cell polarity. Developmental cell *19*, 39-53.

Theveneau, E., Steventon, B., Scarpa, E., Garcia, S., Trepat, X., Streit, A., and Mayor, R. (2013). Chase-and-run between adjacent cell populations promotes directional collective migration. Nature cell biology *15*, 763-772.

Westerfield, M. (2000). The Zebrafish Book. A guide for the laboratory use of zebrafish (Danio rerio). 4th edn. Eugene, OR: University of Oregon Press.

Wang, N., Tolic-Norrelykke, I.M., Chen, J., Mijailovich, S.M., Butler, J.P., Fredberg, J.J., and Stamenovic, D. (2002). Cell prestress. I. Stiffness and prestress are closely associated in adherent contractile cells. American journal of physiology Cell physiology *282*, C606-616.

Wang, X., He, L., Wu, Y.I., Hahn, K.M., and Montell, D.J. (2010). Light-mediated activation reveals a key role for Rac in collective guidance of cell movement in vivo. Nature cell biology *12*, 591-597.

Wu, Y.I., Frey, D., Lungu, O.I., Jaehrig, A., Schlichting, I., Kuhlman, B., and Hahn, K.M. (2009). A genetically encoded photoactivatable Rac controls the motility of living cells. Nature *461*, 104-108.
