# Supplementary figures and images for: Cadherin Switch during EMT in Neural Crest Cells Leads to Contact Inhibition of Locomotion via Repolarization of Forces
Source: Dev Cell. 2015 Aug 24;34(4):421–34. doi: 10.1016/j.devcel.2015.06.012 (PMC4552721; doi:10.1016/j.devcel.2015.06.012)

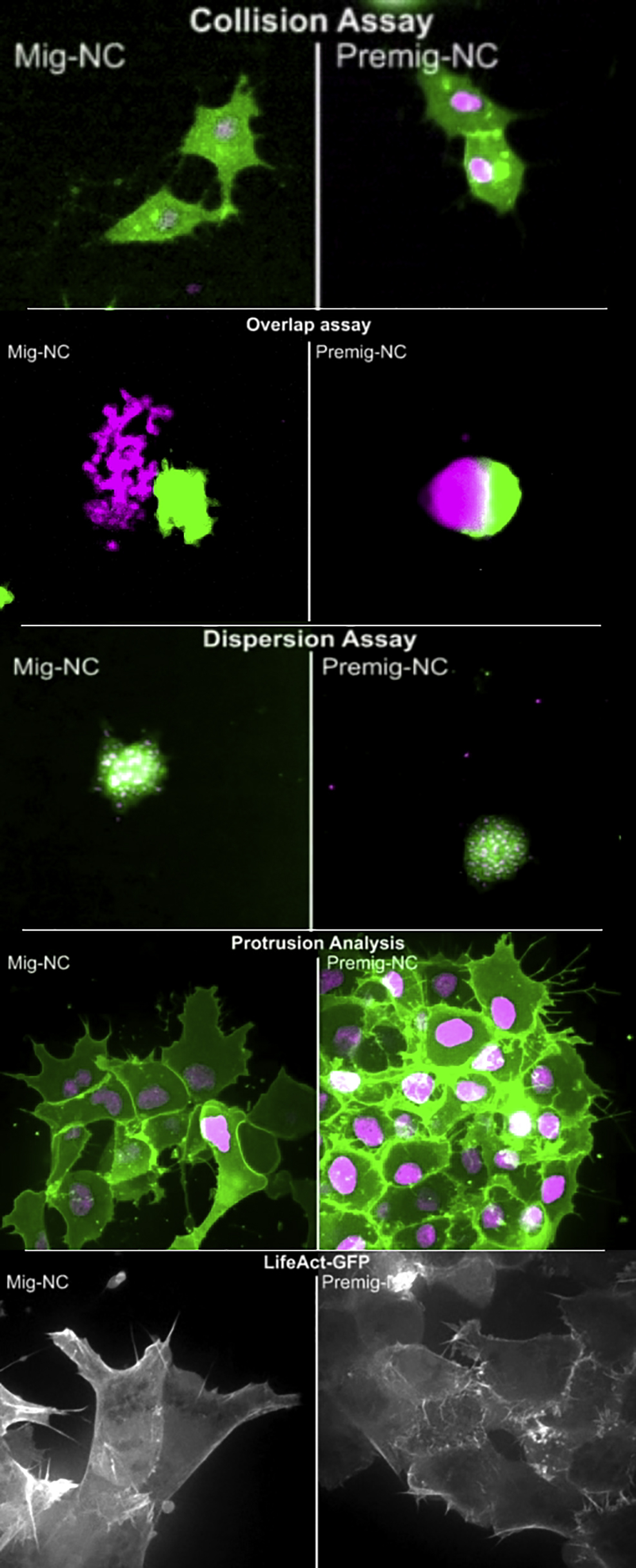

Supplement: Movie S1. CIL Is a Developmentally Regulated Property of NC Cells, Related to Figure 1 — (Collision assay) This is an example of Mig-NC collisions in which cells undergo CIL (left) and of Premig-NC cells forming a stable contact (right). Magenta is nuclear-mCherry. Green is membrane GFP. Frame delay is 5 minutes, and magnification is ×20. (Overlap assay) This is an example of Mig-NC explant overlap assay in which explants undergo CIL and do not overlap (left) and of Premig-NC, in which explants overlap and cells intermingle (right). Magenta is Rhodamine Dextran. Green is fluorescein-dextran. Frame delay is 5 minutes. Magnification is ×10. (Dispersion assay) Mig-NC explants undergo EMT and disperse (left), while Premig-NC does not disperse (right). Magenta is nuclear-mCherry. Green is membrane GFP. Frame delay is 5 minutes. Magnification is ×10. (Protrusion analysis) Mig-NC are strongly polarized and produce protrusions directed outward (arrow, left), while Premig-NC protrusions are not polarized (arrow, right) and produce protrusions at cell-cell contact sites (arrowhead). Magenta is nuclear-mCherry. Green is membrane GFP. Frame delay is 10 seconds. Magnification is ×60. (Lifeact-GFP) Mig-NC are strongly polarized and produce actin-rich protrusions directed outward (arrow, left), while Premig-NC protrusions are not polarized (arrow, right) and produce actin-rich protrusions at cell-cell contacts (arrowhead). Frame delay is 10 seconds, and magnification is ×60. [file mmc2.jpg]

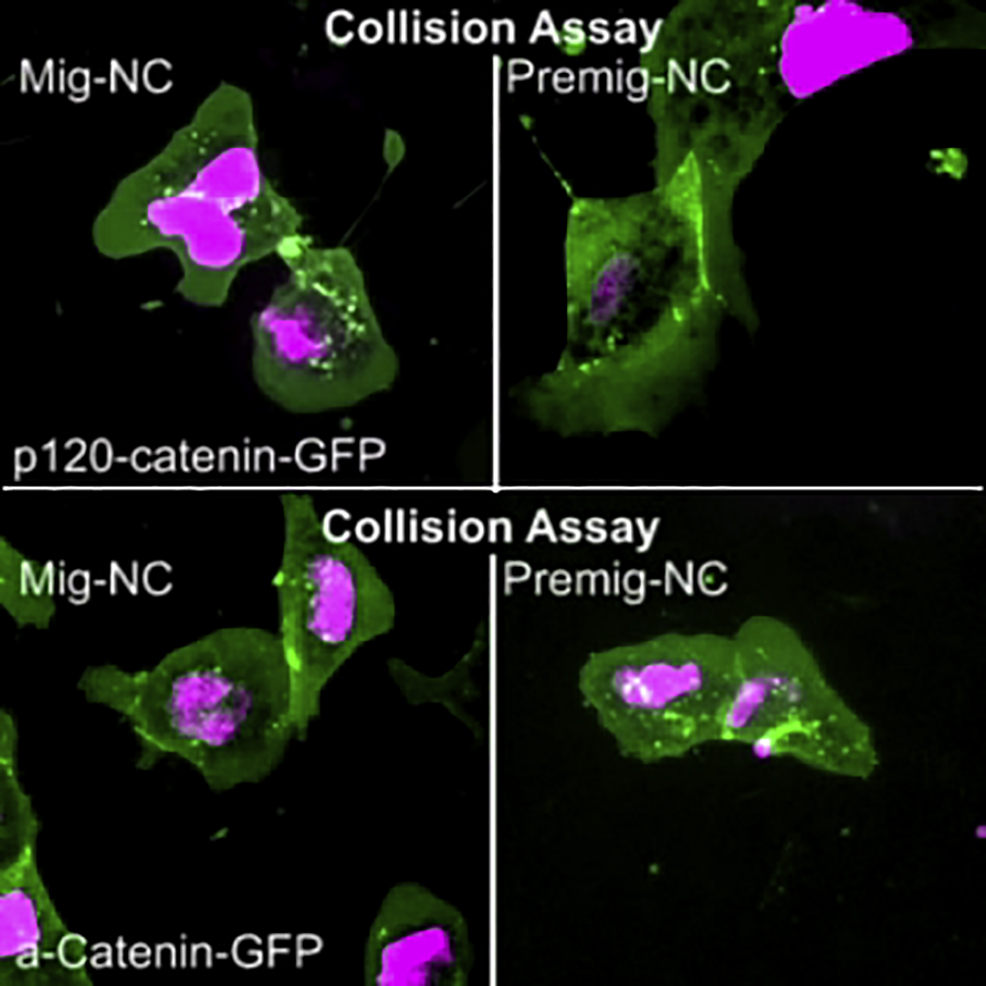

Supplement: Movie S2. Different Dynamics of Junction Disassembly in Migratory and Premigratory NC Cells, Related to Figure 2 — An example of Mig-NC collisions in which cells undergo CIL (left) and of Premig-NC, cells forming a stable junction (right). (Top) p120-GFP (green). (Bottom) α-catenin-GFP (green). Magenta is nuclear-mCherry. Frame delay is 20 seconds. Magnification is ×60. [file mmc3.jpg]

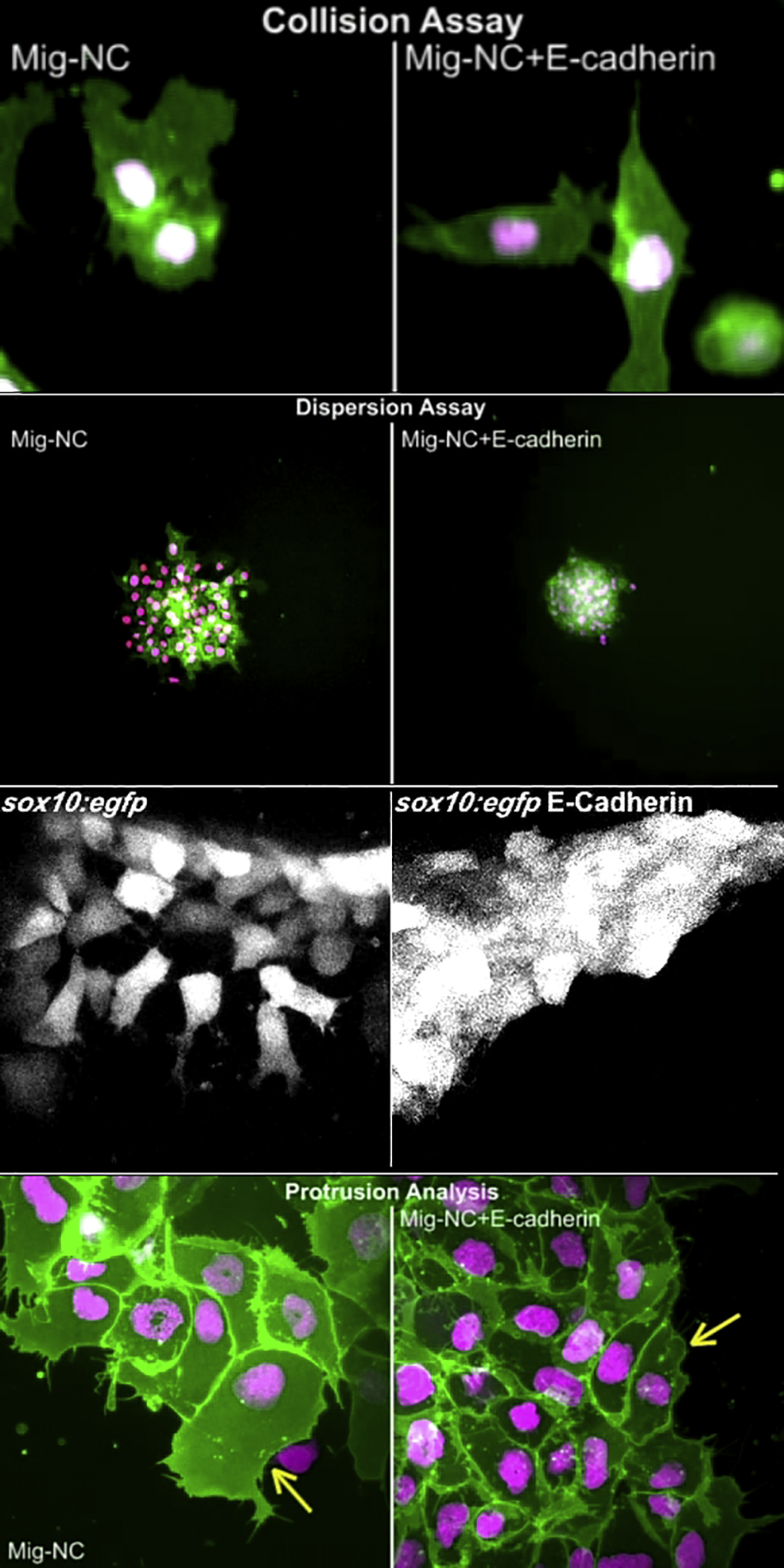

Supplement: Movie S3. E-Cadherin Suppresses CIL In Vivo and In Vitro, Related to Figure 3 — (Collision assay) This is an example of Mig-NC collisions in which cells undergo CIL (left) and of Mig-NC expressing E-cadherin forming a stable contact (right). Magenta is nuclear-mCherry. Green is membrane GFP. Frame delay is 5 minutes. Magnification is ×20. (Dispersion assay) Mig-NC explants undergo EMT and disperse (left), while Mig-NC expressing E-cadherin remains in a more compact cluster (right). Magenta is nuclear-mCherry. Green is membrane GFP. Frame delay is 5 minutes. Magnification is ×10. (Sox10:egfp) Zebrafish embryos. sox10:egfp NC (left) is strongly polarized and produce protrusions directed outward (arrow), while in E-cadherin expressing sox10:egfp embryos (right), NC protrusions are not polarized. Channel is GFP. Frame delay is 4 minutes. Magnification is ×50. (Protrusion analysis) Mig-NC (left) are strongly polarized and produce protrusions directed outward (arrow), while in E-cadherin expressing Mig-NC (right) protrusions are not polarized (arrow) and produce protrusions at cell-cell contact sites (arrowhead). Magenta is nuclear-mCherry. Green is membrane GFP. Frame delay is 10 seconds. Magnification is ×60. [file mmc4.jpg]

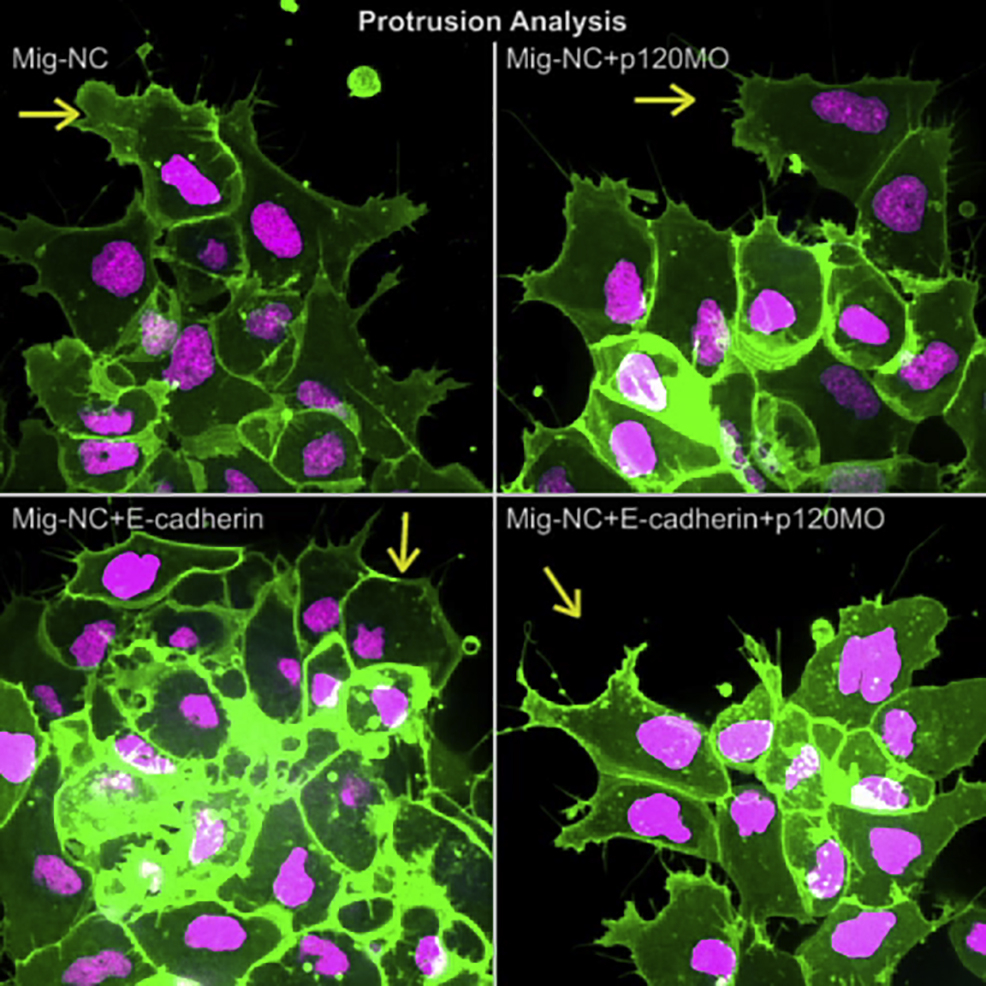

Supplement: Movie S4. p120 Knockdown Rescues Protrusion Formation in E-Cadherin Expressing Migratory NC, Related to Figure 4 — (Protrusion analysis) Mig-NC (top left) or Mig-NC +p120 MO (top right) are polarized and produce protrusions directed outwards (arrow), while in E-cadherin expressing Mig-NC, protrusions are not polarized (arrow, bottom left). P120 knockdown restores normal protrusion polarity in E-cadherin expressing NC (arrow, bottom right). Magenta is nuclearmCherry. Green is membrane GFP. Frame delay is 10 seconds. Magnification is ×60. [file mmc5.jpg]

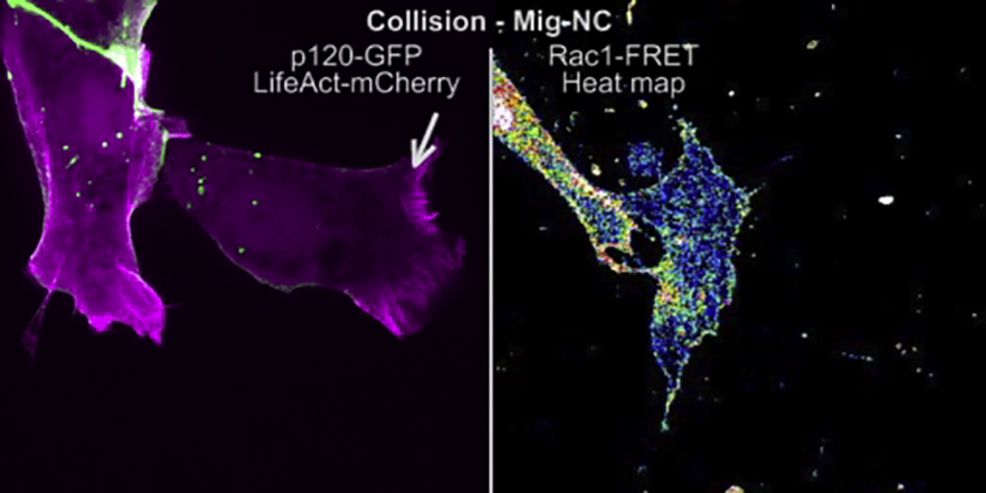

Supplement: Movie S5. Repolarization of Protrusions and of Rac1 Activity Precedes Junctional Disassembly during CIL, Related to Figure 5 — (Mig-NC cell-cell collision) p120-GFP (green) and lifeactmCherry (magenta) are in left, and Rac1 FRET (heatmap) is in right. Protrusions formation (arrow, left) and Rac1 activation (arrow, right) at the free edge correlate with the disassembly of the p120- GFP positive cell-cell junction (arrowhead, left). Frame delay is 1 minute. Magnification is ×60. [file mmc6.jpg]

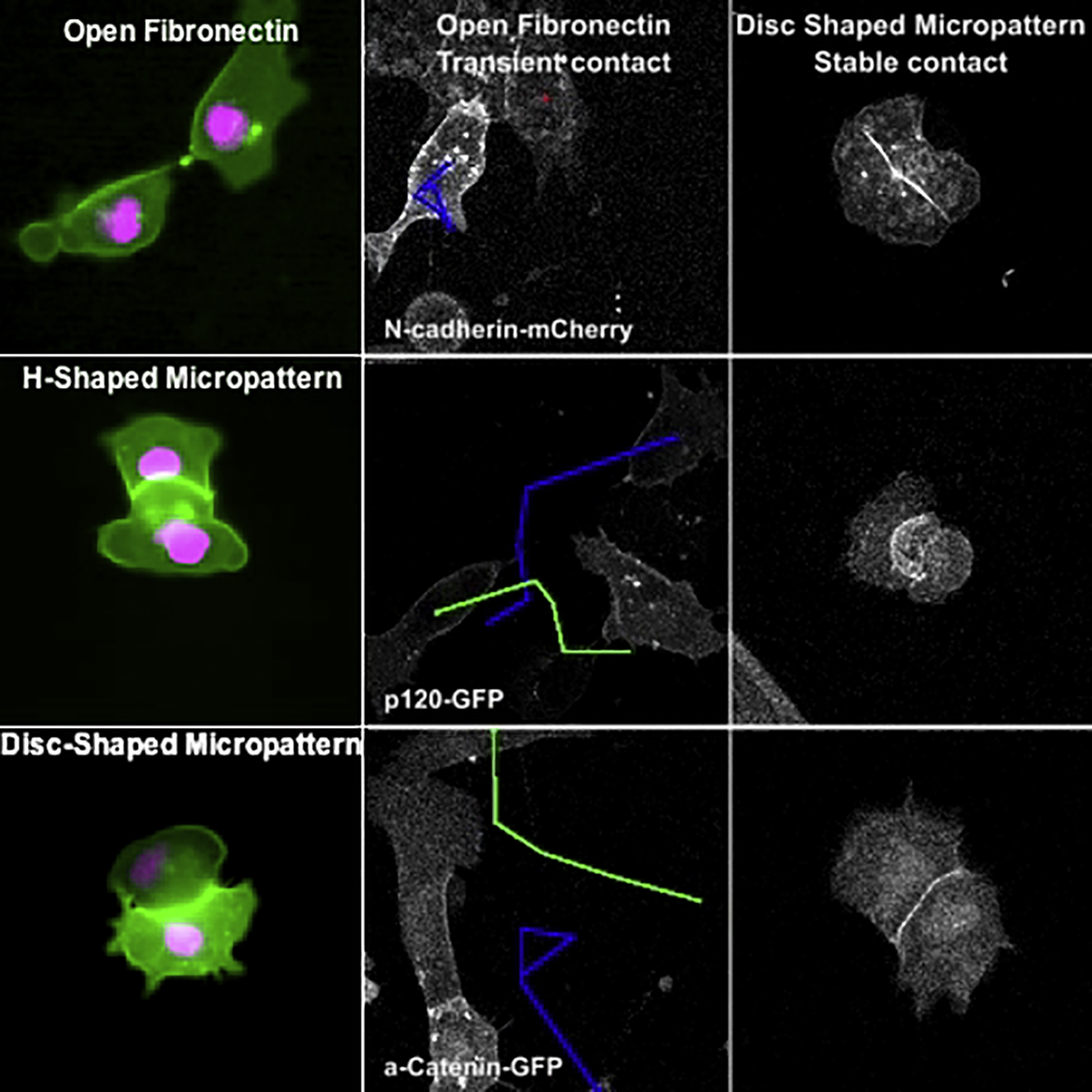

Supplement: Movie S6. Cell Confinement Inhibits CIL, Related to Figure 5 — (Left column) Cell confinement inhibits CIL. This is an example of Mig-NC collisions in unconstrained (top) Fn and of Mig-NC confined in a H-shaped (middle) or disc-shaped micropattern (bottom). Magenta is nuclear-mCherry. Green is membrane GFP. Frame delay is 5 minutes. Magnification is ×60. (Central column) Mig-NC on unconstrained FN; (top) NCadherin-mCherry; (middle) p120-GFP; (bottom) α-catenin-GFP injected NC. (Right column) Mig-NC confined on a disc micropattern, cells forming a stable junction; (top) N-cadherin-mCherry; (middle) p120-GFP; (bottom) α-catenin-GFP injected NC. Frame delay is 3 minutes. Magnification is ×60. [file mmc7.jpg]

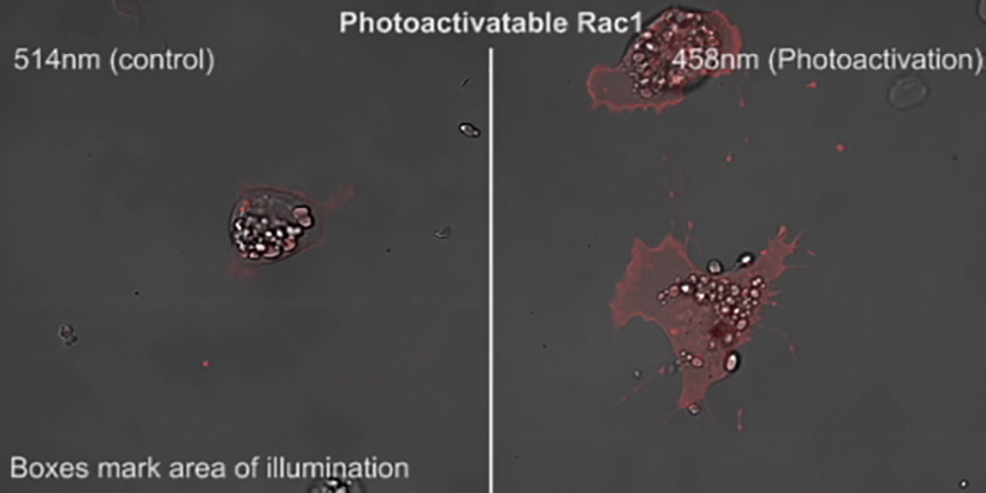

Supplement: Movie S7. Protrusion Repolarization via Rac1 Is Sufficient to Trigger Cell Separation during CIL, Related to Figure 6 — (Photocativation of Rac1 on single cells) PA-Rac promotes protrusion formation in single NC cells. The boxed area was illuminated with 45-second pulses of 514 nm control laser light (left) or with 458 nm wavelength (right). Red is PA-Rac-mCherry Grey is transmitted light. Frame delay is 1 minute. Magnification is ×60. (Photoactivation of dom-neg Rac1 on single cells) DN-PA-Rac promotes protrusion collapse in single NC cells. The boxed area was illuminated with 45-second pulses of 514 nm control laser light (left) or with 458 nm wavelength (right). Red is DN-PA-Rac-mCherry. Grey is transmitted light. Frame delay is 1 minute. Magnification is ×60. (Photoactivation of dom-neg Rac1 on pair of cells) DN-PA-Rac inhibits cell dissociation in Mig-NC doublets. The boxed areas were illuminated with 45-second pulses of 514 nm control laser light (left) or with 458 nm wavelength (right). Red is PA-Rac-mCherry Green is membrane-GFP. Frame delay is 1 minute. Magnification is ×60. (Photoactivation of Rac1 on pair of cells overexpressing E-cadherin) PA-Rac promotes cell dissociation in E-cadherin expressing NC doublets. The boxed areas were illuminated with 45-second pulses of 514 nm control laser light (left) or with 458 nm wavelength (right). Red is PA-Rac-mCherry. Green is E-cadherin-GFP. Frame delay is 1 minute. Magnification is ×60. (Photoactivation of Rac1 on pair of cells overexpressing E-cadherin-GFP) PA-Rac activation by illumination does not affect E-cadherin junctional recruitment. E-cadherin-GFP is injected Mig-NC cells. The boxed areas were illuminated with 45-second pulses of 514 nm control laser light (left) or with 458 nm wavelength (right). Frame delay is 1 minute. Magnification is ×60. [file mmc8.jpg]
